# Genome-wide analysis and expression pattern of the *ZoPP2C* gene family in *Zingiber officinale* Roscoe

**DOI:** 10.1186/s12864-024-09966-w

**Published:** 2024-01-20

**Authors:** Pan Zhang, Deqi Liu, Jiawei Ma, Chong Sun, Zhaofei Wang, Yongxing Zhu, Xuemei Zhang, Yiqing Liu

**Affiliations:** 1 College of Horticulture and Gardening, Spice Crops Research Institute, Yangtze University, Jingzhou, 434025 Hubei China; 2 Special Plants Institute, College of Landscape Architecture and Life Science, Chongqing University of Arts and Sciences, Chongqing, 402160 China

**Keywords:** *PP2C* gene family, Ginger, Genome-wide analysis, Chilling stress, Functional analysis

## Abstract

**Background:**

Protein phosphatases type 2C (*PP2C*) are heavily involved in plant growth and development, hormone-related signaling pathways and the response of various biotic and abiotic stresses. However, a comprehensive report identifying the genome-scale of *PP2C* gene family in ginger is yet to be published.

**Results:**

In this study, 97 *ZoPP2C* genes were identified based on the ginger genome. These genes were classified into 15 branches (A-O) according to the phylogenetic analysis and distributed unevenly on 11 ginger chromosomes. The proteins mainly functioned in the nucleus. Similar motif patterns and exon/intron arrangement structures were identified in the same subfamily of ZoPP2Cs. Collinearity analysis indicated that ZoPP2Cs had 33 pairs of fragment duplicated events uniformly distributed on the corresponding chromosomes. Furthermore, ZoPP2Cs showed greater evolutionary proximity to banana’s PP2Cs. The forecast of *cis*-regulatory elements and transcription factor binding sites demonstrated that *ZoPP2Cs* participate in ginger growth, development, and responses to hormones and stresses. *ZoERFs* have plenty of binding sites of *ZoPP2Cs*, suggesting a potential synergistic contribution between *ZoERFs* and *ZoPP2Cs* towards regulating growth/development and adverse conditions. The protein–protein interaction network displayed that five ZoPP2Cs (9/23/26/49/92) proteins have robust interaction relationship and potential function as hub proteins. Furthermore, the RNA-Seq and qRT-PCR analyses have shown that *ZoPP2Cs* exhibit various expression patterns during ginger maturation and responses to environmental stresses such as chilling, drought, flooding, salt, and *Fusarium solani*. Notably, exogenous application of melatonin led to notable up-regulation of *ZoPP2Cs* (17/59/11/72/43) under chilling stress.

**Conclusions:**

Taken together, our investigation provides significant insights of the ginger *PP2C* gene family and establishes the groundwork for its functional validation and genetic engineering applications.

**Supplementary Information:**

The online version contains supplementary material available at 10.1186/s12864-024-09966-w.

## Background

Reversible protein phosphorylation is a well-known mechanism that regulates multiple physiological responses in plants. Protein kinases (PKs) mainly catalyze protein phosphorylation, while the protein phosphatases (PPs) catalyze the dephosphorylation of phosphorylated proteins [1]. In the process of life activities, PPs and PKs mainly regulate the activity and function of proteins through reversible phosphorylation of proteins, and ultimately activate or inhibit the activity of the corresponding enzymes [2–4], thus affecting the cell cycle, hormone response, and metabolic regulation and other life activities [5, 6]. PPs can be classified into three groups based on their substrate specificity: serine/threonine protein phosphatases (STPs), tyrosine protein phosphatases (PTPs), and dual-specificity protein phosphatases (DSPTPs) [6]. Based on the analysis of the molecular biological structure, STPs can be categorized into two families: phosphoprotein phosphatase (PPP), including PP1, PP2 and PP3. The primary function of PP2 is to catalyse the dephosphorylation of the α-subunit of phosphorylated kinases. PP2 can be subdivided into three classes, PP2A, PP2B, and PP2C, based on substrate specificity and sensitivity to inhibitors [5, 7].

The PP2C family is the biggest PP family in plants [8]. It has been shown that PP2C plays an important role in precisely regulating the cellular pathway of plant protein phosphorylation. In contrast to the PPP family, the PP2C family does not include regulatory subunits, it has distinct structures that can bind to the structural domains of catalytic phosphatases. More than 80 members of the *PP2C* have been identified in *Arabidopsis thaliana* [9], rice [10], soybean [11], alfalfa [12], maize [13], and cotton [14]. The *PP2Cs* are reported to regulate plant growth and development processes, such as root growth, organ initiation, stem cell polarity, seed dormancy, and cell expansion [15]. Furthermore, *PP2C* members are capable of responding to a range of biotic and abiotic stressors, including fungi, wounds, drought, salt, and cold [16, 17]. Besides, hormonal pathways such as the abscisic acid (ABA) [15], jasmonic acid (JA) [18] and salicylic acid (SA) [19] pathways are highly dependent on *PP2C* members. Currently, numerous studies have concentrated on the involvement of *PP2C* members in abiotic stress [7]. In rice, the *OsSIPP2C1* can respond to salt and drought stresses, and the accumulation of *OsSIPP2C1* has a negative effect on the ABA signaling pathway, during the later stages of spike development, the decrease of *OsSIPP2C1* can ensure ABA facilitates rice spike maturation [20]. *TaPP2C1* exhibited reduced expression levels during treatments with both ABA and NaCl in wheat [21]. While overexpression of *TaPP2C1* in tobacco resulted in increased salt resistance, decreased accumulation of reactive oxygen species, and upregulation of genes involved in the ABA-independent pathway [22]. Furthermore, *PP2Cs* have been found to contribute to plant resistance against chilling stress. For example, overexpression of maize *ZmPP2C2* exhibited higher germination rates, increased activities of antioxidant enzymes and greater tolerance to cold stress, which suggested that *ZmPP2C2* may act as a positive regulator of plant resistance to chilling stress [23]. Although the *PP2Cs* have been widely known to play crucial roles in plant development and coping with environmental stresses in many plant species [15], there is a lack of comprehensive identification and analysis of the *PP2Cs*’ expression patterns under stresses in ginger (*Zingiber officinale* Roscoe).

Ginger is a valuable medicinal plant, containing 6-gingerol and curcuminoids compounds, with antioxidant, antimicrobial and anti-inflammatory properties [24, 25]. It is also common used as flavouring and widely cultivated worldwide [26]. Environmental stresses have considerable impacts on ginger growth and development. For example, ginger is susceptible to sunburn at high temperature and growth reduction in low temperature [27]. Ginger can withstand a certain degree of drought; however, long-term drought has a significant inhibitory effect on rhizome growth, resulting in reduced yields. Ginger seedlings often suffer from severe salt damage [28]. To breed new varieties, it is necessary to investigate the developmental mechanisms and abiotic tolerance of ginger. In this study, 97 *ZoPP2C* genes were identified from the ginger genome. Using bioinformatics, the gene structure, motif composition, evolutionary relationships, chromosomal localization, covariance analysis and protein interactions network were analyzed. Additionally, we explored the expression profiles of *ZoPP2Cs* in the different tissues of gingers, the different growth stages, and under stressful conditions. These findings would provide significant insights into the functional attributes of *PP2C* family genes, thereby supporting genetic enhancement efforts of ginger.

## Result

### Identification and physicochemical characterization of *ZoPP2C* genes in ginger

The PP2C family (PF00481) was queried using the Pfam database (http://pfam.xfam.org/) and 128 PP2C candidate genes were obtained by Hidden Markov Model (HMM) approach using a threshold selection of e < 10^–5^. To validate the identified candidates, the obtained 128 protein sequences were assembled and screened against the NCBI conserved structural domain database (https://www.ncbi.nlm.nih.gov/Structure/bwrpsb/bwrpsb.cgi) and the SMART database (http://smart.embl-heidelberg.de/), which were combined with the available whole genome data of ginger. 31 incorrectly models that did not have PP2C conserved structural domains were excluded, and 97 ginger *ZoPP2C* genes were finally identified for further study (Table S1).

The detailed physiological and biochemical information including the amino acid length (AA), molecular mass (MW), isoelectric point (PI), hydrophilicity (dGRAVY), instability index, and predicted subcellular localization of ZoPP2Cs was analyzed, and listed in Table S2. The results showed that the amino acid lengths of 97 ZoPP2Cs ranged from 273 AA (ZoPP2C37) to 1068 AA (ZoPP2C79), the relative molecular masses were concentrated between 30.18 kDa (ZoPP2C40) and 118.73 kDa (ZoPP2C79). The important physico-chemical properties of isoelectric points ranged from 4.67 (ZoPP2C19) to 9.17 (ZoPP2C45). In addition, the hydrophilicity of ZoPP2C family members ranged from -0.562 (ZoPP2C84) to -0.01 (ZoPP2C41), indicating that ZoPP2Cs are hydrophilic proteins. Among 97 ZoPP2Cs, the instability indices of 17 proteins were lower than 40, suggesting that the majority of ZoPP2Cs are unstable proteins. According to the online software of BUSCA, 68 ZoPP2Cs were predicated to localize in the nucleus, 20 ZoPP2Cs in the chloroplast, 4 ZoPP2Cs in the extracellular, 3 ZoPP2Cs in the mitochondrion, 1 ZoPP2C in the cytoplasm, and 1 ZoPP2C in the endomembrane.

### Structural characterization and phylogenetic analysis of the ZoPP2Cs

**Fig. 1:**
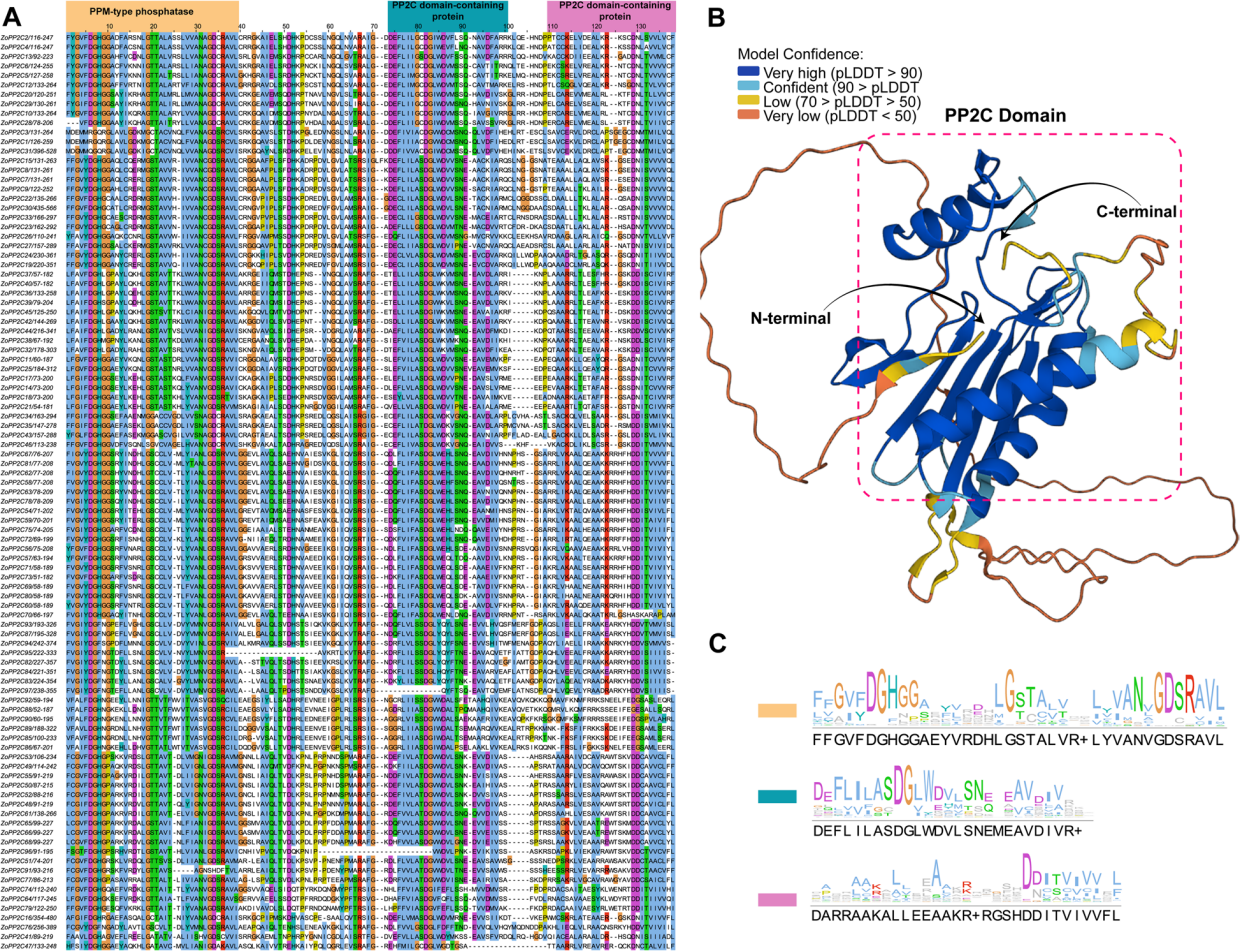
Structural features of the ZoPP2Cs. **A** Multiple sequences alignment of ZoPP2C conserved domains. **B** 3D structural model of PP2C structural domains. **C** ZoPP2C sequence logos for conserved domains, generated using Jalview (v2.10.3). The height of the symbols within the stack indicates the relative frequency of each amino acid. Logos were created by comparing multiple ZoPP2C protein sequences

According to the Interpro (https://www.ebi.ac.uk/interpro/) analysis, four major ZoPP2C protein structural domains including PPM-type phosphatase-like domain, PP2Cc, PP2C-SIG, PP2C_like were identified in 97 ZoPP2Cs’ amino acids. Multiple sequence comparisons showed that most of the core sequences in 97 ZoPP2C proteins contained complete or nearly complete structural domains, only individual amino acids in the conserved motifs had specific mutations or evolution in some ZoPP2C members (Fig. 1A). Figure 1A shows that all 97 ZoPP2C proteins contain PPM-type phosphatase-like structural domains and PP2C domain-containing protein. Although individual members such as ZoPP2C84, ZoPP2C95 and ZoPP2C96 have considerable amino acid sequence variation, a search for functional structural domains on SMART confirms that these genes still belong to the PP2C family. Based on the established three-dimensional structure of PP2C, it can be seen that the conserved domains of ZoPP2Cs are mainly concentrated in the PP2C Domain, which generally has an extension region at the N-terminal or C-terminal (Fig. 1B). Logo represented the conservation of partial structural domain sequences (Fig. 1C). The height of the symbols within the stack indicated the relative frequency of each amino acid.

**Fig. 2:**
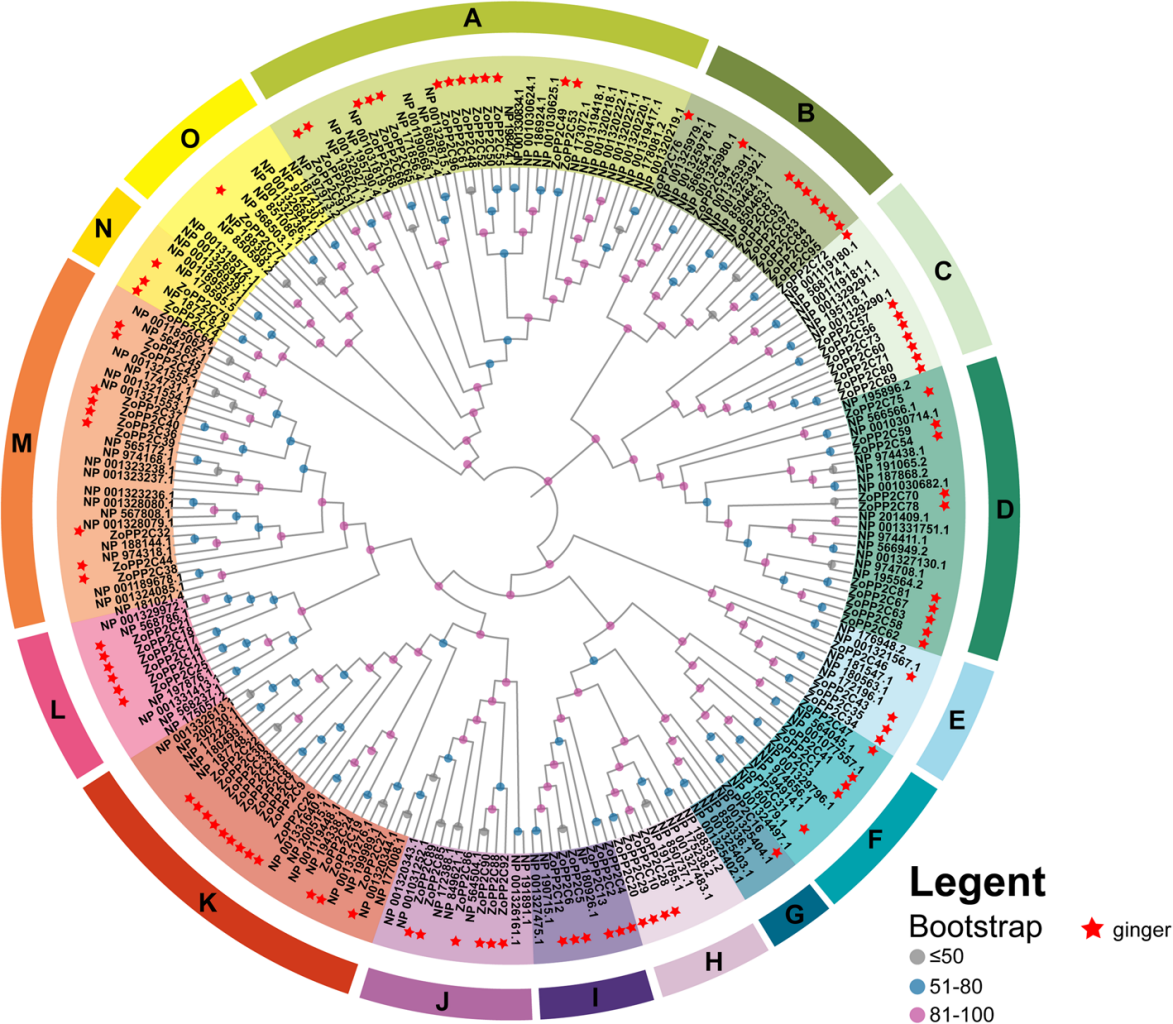
Phylogenetic analysis of the PP2C genes. Phylogenetic trees for A. thaliana and ginger PP2C gene families were modelled using maximum likelihood (ML) and 1000 bootstrap replicates were selected. Bootstrap levels are indicated by different colored circles. Red stars specifically indicate the ginger ZoPP2C sequences

The phylogenetic relationship of *PP2C* genes between ginger and model plants was further investigated, and we constructed a phylogenetic tree based on 134 *A. thaliana* and 97 ginger *PP2C* genes using the maximum likelihood method (ML) (Fig. 2). The 97 ZoPP2Cs were classified into 15 branches (A-O), and each branch contained a widely varying number of PP2C members. In ginger, 13, 9, 8, 10, 4, 5, 4, 6, 6, 12, 6, 9, 3 ZoPP2Cs were distributed in A, B, C, D, E, F, H, I, J, K, L, M, N, respectively, whereas there was only one ZoPP2C in subfamilies G and O, respectively. Phylogenetic analyses showed that each subfamily contained PP2C proteins from both ginger and *A. thaliana*, which tended to form small independent branches in each subgroup. The results suggests that the *PP2C* gene family may evolve from different ancestors. According to the chromosomal localization analysis, as it is shown in Fig. S1, 97 ZoPP2C genes were located on 11 chromosomes, of which 7, 3, 6, 12, 14, 11, 10, 4, 8, 6 and 16 *ZoPP2Cs* were located on chromosome chr02 (chromosome 1) to chr22 (chromosome 11). The results showed that *ZoPP2C* genes were widely distributed on 11 ginger chromosomes, but unevenly distributed.

### Gene structure and motif composition of the ZoPP2Cs

**Fig. 3:**
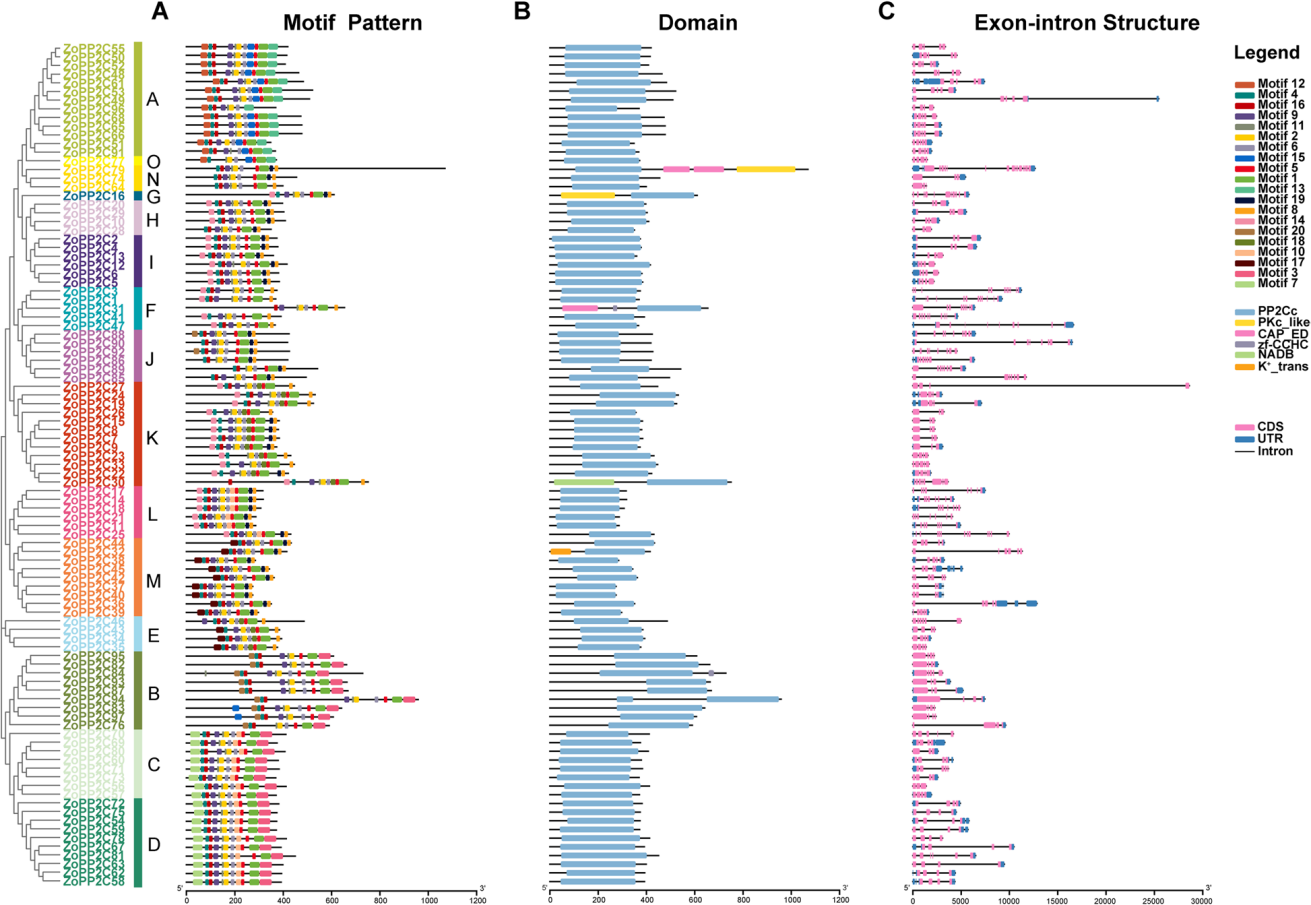
Phylogenetic relationships and gene structure of the ZoPP2Cs. **A** The Motif pattern of ZoPP2Cs. The protein was comprised of motifs labelled 1–20 and featured in varied, colored boxes. Sequences for each motif can be found in Fig. S2. Phylogenetic analysis was conducted on the complete sequence of the ginger ZoPP2C proteins using MEGA X software. **B** PP2C domains of ZoPP2Cs. The blue color represented the PP2C domain. **C** The exon–intron structures of ZoPP2Cs. Blue boxes indicated the 5' and 3' untranslated regions, while red boxes indicated the exons. Black lines represent the introns. The length of the protein can be estimated using the scale located at the bottom

To clearly reveal the structural features of ginger ZoPP2C members, we analyzed the conserved motifs and gene structures based on phylogenetic relationships (Fig. 3). For ZoPP2C proteins, 20 conserved motifs were identified using MEME. Motifs 1, 2, 4, 5, 6, 9, 11, 12, 13, 15, 16, and 19 existed in almost all ZoPP2C proteins, while members of subfamilies C and D also had structural features of Motifs 3, 7, and 10. Motif 14 was identified in subfamilies G, H, I, F, K and L, whereas motif 17 was present in subfamilies M and E. Similarly, motif 18 was distributed among subfamilies K and E, whereas motif 20 was exclusively detected in subfamily B (Fig. 3A). This suggests that all identified PP2Cs have typical family characteristics, especially in the same subgroup. the conserved domains of ZoPP2C proteins were analyzed using NCBI online tool (Fig. 3B), and the results showed that ZoPP2Cs mainly contained PP2Cc feature region, while individual family members such as ZoPP2C79 and ZoPP2C16 contained PKc and CAP_ED domains (effector domain of the CAP family of transcription factors), and ZoPP2C30, ZoPP2C32 contained NADB, K^+^_trans conserved domains, respectively. In addition, the exon–intron organization of these genes was tested (Fig. 3C), 69 *ZoPP2C* genes had the untranslated region (UTR), while 28 genes were without untranslated region. 97 ZoPP2Cs had introns which varies from 3 to 16. The members in some subgroups such as B, K, O, and N had similar exon/intron structures including its number of introns, exon lengths, and positions. However, the number and length of introns were completely different in different subfamilies, such as I, F, and K. These results suggest that the diversity in the gene structure of *ZoPP2Cs* may play an important role in the functional divergence of its evolutionary process.

### Collinearity and synthesis analysis of ZoPP2Cs

**Fig. 4:**
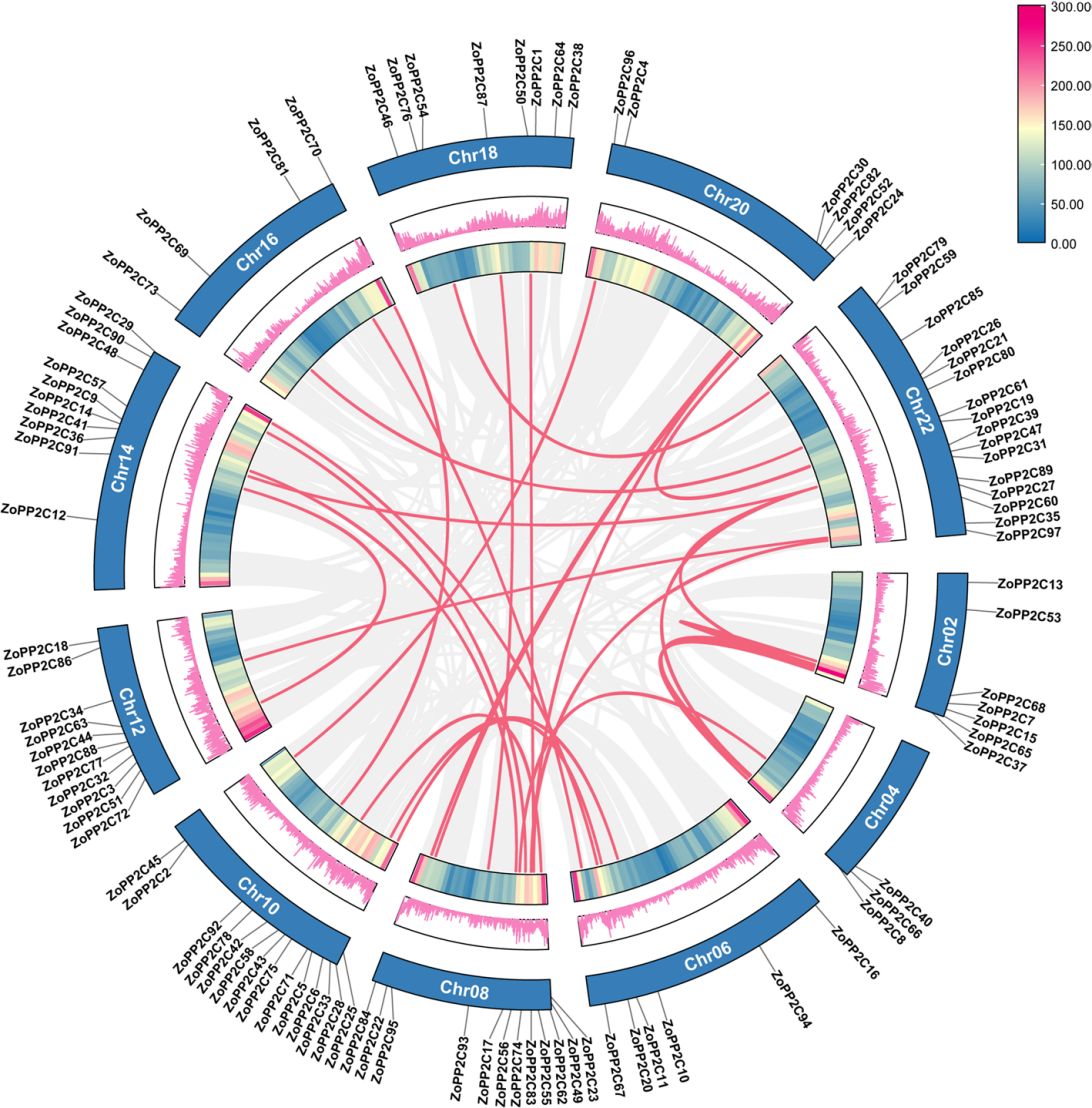
Schematic diagram of ZoPP2Cs collinearity in ginger. The red lines indicated ZoPP2C gene pair duplications in ginger

Gene duplication plays an important role in the generation of new functions and the expansion of gene families. Closely related genes with a distance of less than 200 kb on the same chromosome are defined as tandem duplications, otherwise as fragment duplications [29]. To further understand the amplification mechanism of ZoPP2Cs, we analyzed the fragment and tandem repeats of ZoPP2Cs in the ginger genome by MCscan. The results showed that no tandem duplication events were found in the PP2C gene family, but 33 pairs of fragment duplication events were identified and distributed on the corresponding chromosomes (Fig. 4). Among these 33 co-linkage pairs, 17 pairs of genes showed one-to-many pairings, such as ZoPP2C74-ZoPP2C41 and ZoPP2C74-ZoPP2C90, ZoPP2C82-ZoPP2C22 and ZoPP2C82-ZoPP2C95, suggesting that many of the *ZoPP2Cs* are homologous genes. The ratio of Ka and Ks which is considered as an important reference for species selection evolution were computed, and the results showed that the fragmental duplicates had Ka/Ks ratios fall into the range of 0.1917 to 0.4646 (Table S3), indicating that these genes have been subjected to purification selection in the process of evolution.

### Evolutionary analysis of *ZoPP2C* genes

**Fig. 5:**
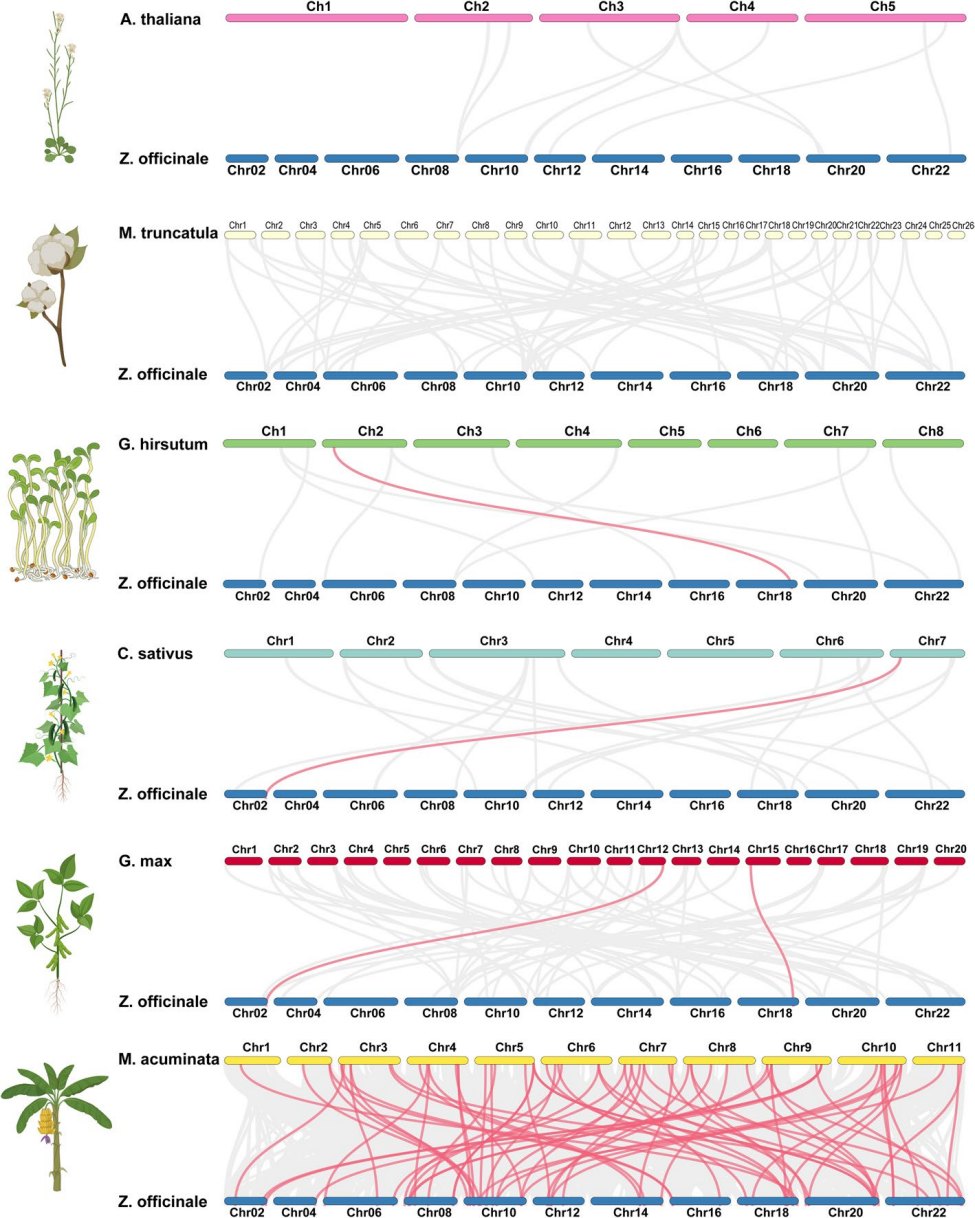
Collinearity analysis of PP2C genes in ginger and 6 representative plants. The grey lines in the background illustrated the blocks of ginger that co-localize with other plant genomes, whereas the red lines represented pairs of co-linear PP2C genes

To investigate the evolutionary relationship of the PP2C family, we analyzed the covariance of PP2C genes between ginger and six plants, including *A. thaliana*, cotton, alfalfa, cucumber, soybean and banana. As can be seen in Fig. 5, there was no covariance between *A. thaliana*, cotton and ginger, and only 1–2 pairs of homologous genes existed between alfalfa, cucumber and soybean, while 69 repetitive events were identified between banana and ginger and the collinearity blocks were mainly distributed on the chr8, 10, 18, 20 and 22. Multiple ginger *ZoPP2C* genes are homologous to a single banana *PP2Cs* gene, and multiple banana *PP2Cs* genes are also homologous to a single ginger *ZoPP2C* gene (Table S4). This suggests that some of the *ZoPP2C* genes may have existed before the species diverged, and that the PP2Cs in banana are phylogenetically closer to the ZoPP2Cs in ginger since banana is a closely related monocotyledon with ginger.

### Prediction of *cis*-acting regulatory elements

**Fig. 6:**
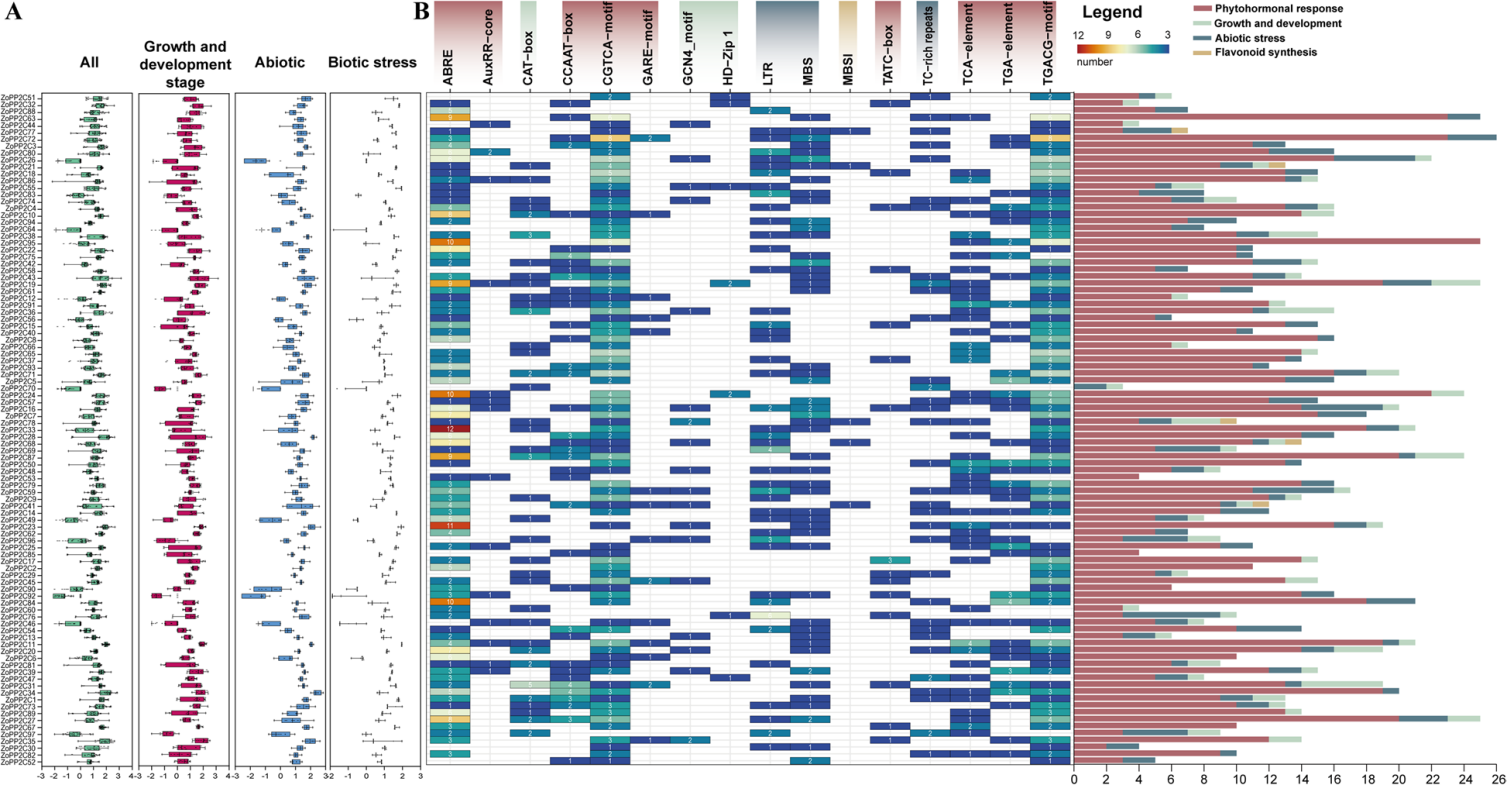
Cis-regulatory elements of the ZoPP2C promoters. **A** Box plots of ZoPP2C promoters. Box plots depicted the expression levels of ZoPP2Cs during various growth stages under different stress conditions. **B** Heatmap of the ZoPP2C promoters. Heatmap displayed the number of cis-elements, whereby larger numbers were highlighted in red and smaller numbers in blue. Histograms exhibited the proportion of varied cis-elements, with diverse colors denoting distinct types of cis-elements

To better understand the functions of ZoPP2Cs, especially under abiotic stress conditions and in relation to plant endogenous hormone responses, we extracted upstream 2 kb sequence from each *ZoPP2C* genes to predict *cis*-regulatory elements by the PlantCARE database (Fig. 6). The results showed that the promoter sequences of ZoPP2Cs had a variety of *cis*-regulatory elements which could be classified into four categories (Fig. 6A). The hormone related elements like ABRE, AuxRR-core, CCAAT-box, CGTCA-motif, TGACG-motif were widely presented in all promoter regions, suggesting that ZoPP2Cs were involved in the regulation of multiple plant hormone responses.Various of elements related to abiotic stresses were also identified, for example, LTR and MBS were reported to respond to chilling stress, TC-rich repeats elements worked in drought and salt stress responses. What’s more, CAT-box, GCN4-motif, and HD-zip response elements were identified to function in plant growth and development. MBSI element was found to mainly act in the process of flavonoid synthesis. Based on the expression profiles of 97 *ZoPP2Cs* at growth and developmental stages as well as under abiotic and biotic stresses (Fig. 6A), it was shown that *ZoPP2C* genes with higher expression profiles tended to usually contain a higher number of cis-regulatory elements in their promoter regions, and that the number of cis-regulatory elements may play an important role in the expression regulation of ZoPP2Cs. In addition, statistics on the number of these four categories showed that the *ZoPP2C* response components were principally dominated by hormone synthesis, occupying the largest number of components (Fig. 6B), followed by abiotic stress, plant growth and development, suggesting that *ZoPP2Cs* play important roles in the regulation of endogenous hormones and abiotic stresses in plants.

### Protein–protein interaction network construction and prediction of upstream transcription factors

**Fig. 7:**
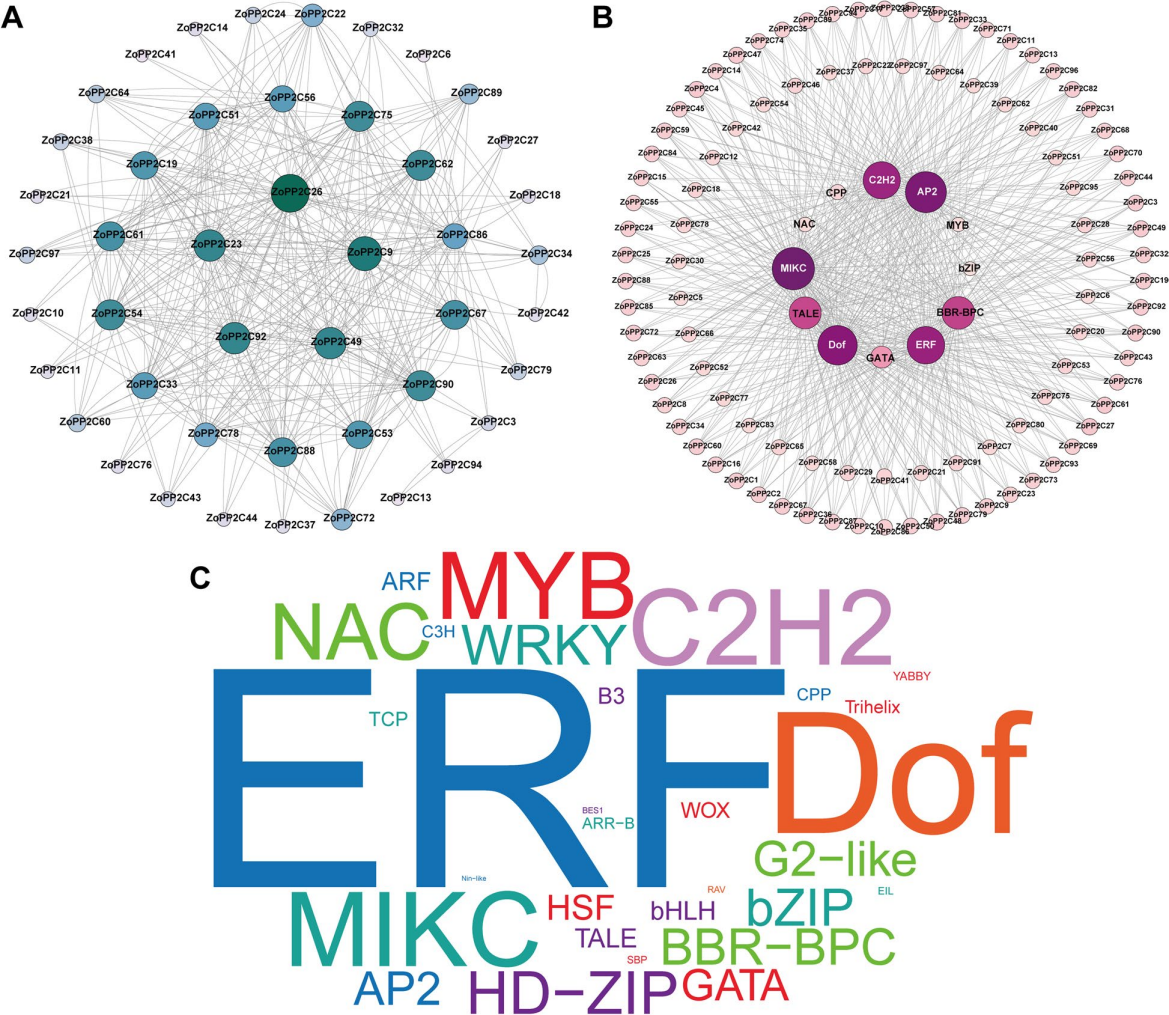
Network analysis of protein–protein interactions and upstream regulatory transcription factors. **A** ZoPP2Cs protein interaction network. The darker and larger circles indicated the proteins with higher connectivity degree. **B** Upstream TFs regulatory network. **C** Word cloud. Word size was positively correlated with the number of corresponding ZoPP2Cs binding sites

In order to investigate the interaction relationships between individual members of the ZoPP2C family, the ZoPP2C protein interaction network was analyzed using online software String, and the connectivity degree of each ZoPP2Cs in the interaction network was analyzed. The degree of connectivity represents the sum of all other proteins connected by a given one. In PPI networks, the 97 ZoPP2Cs were divided into three tiers which contained 97 nodes and 463 edges according to the degree of connectivity. As was shown in Fig. 7A, the innermost circle contains 5 members, namely ZoPP2C9, ZoPP2C23, ZoPP2C26, ZoPP2C49, and ZoPP2C92, with a connectivity degree of 32–42; and the second circle contains 14 ZoPP2Cs with a connectivity degree ranging from 20 to 30, and the outermost circle contains 78 ZoPP2Cs with a connectivity degree less than 20 (Table S5). The KEGG enrichment analysis revealed that ZoPP2C9, ZoPP2C23, ZoPP2C26, ZoPP2C49 and ZoPP2C92 were involved in phenylpropanoid biosynthesis, the MAPK signaling pathway, and the phytohormone signaling pathway (Table S6). Taken together, the PPI network analysis further suggests that ZoPP2Cs proteins may be involved in multiple physiological pathways through protein interactions.

Transcription factors (TFs) are proteins that regulate gene expression at the transcriptional level through specific DNA-binding domains, ensuring that the target gene is expressed in a specific manner. According to PlantTFDB searching and analysis, a total of 36 different TF families were identified to regulate ZoPP2Cs, including MIKC, DOF, ERF, AP2, C2H2, TALE, BBR-BPC, MYB, NAC, WRKY, and et al. (Table S7). As can be derived by constructing the network of ZoPP2Cs regulating by potential TFs (Fig. 7B), MIKC had the highest degree (90), followed by AP2 (86), DOF (81), ERF (75), C2H2 (74), BBR-BPC (60), TALE (58), and GATA (26). In contrast, the CPP, NAC, MYB, and bZIP families had lower degrees ranging from 1 to 5. In addition, we counted the top 30 TF gene families with the most binding sites to ZoPP2C proteins, including ERF, Dof, C2H2, MIKC, MYB, NAC, HD-ZIP, and WRKY (Fig. 7C, Table S8). Overall, the predicted regulatory network suggests that these transcription factor families are significantly enriched in ZoPP2C and may play an important role in regulating ZoPP2Cs proteins in ginger.

### GO analysis of ZoPP2C proteins

**Fig. 8:**
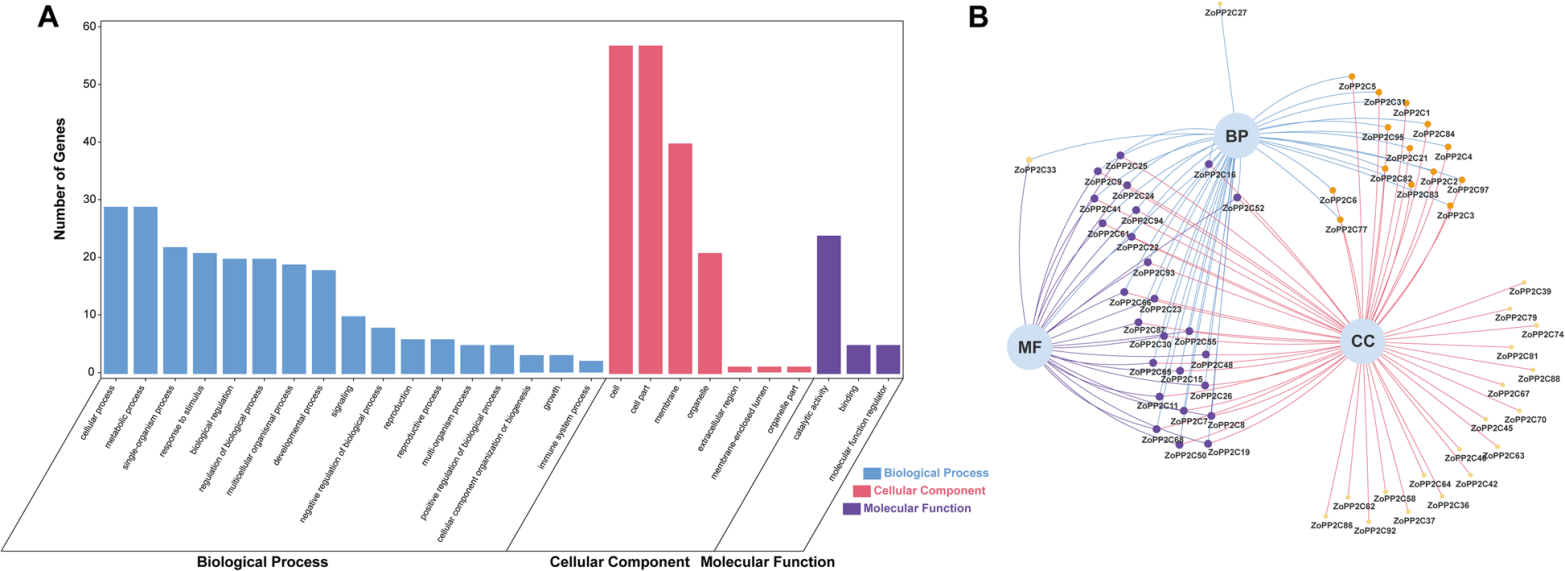
Functional classification of the ZoPP2Cs based on gene ontology (GO). **A** GO analysis of ZoPP2Cs. Visualization showed 27 GO terms at the molecular function (MF), biological process (BP) and cellular component (CC) levels. X-axis indicated the information of GO terms and y-axis indicated the number of enriched genes. **B** Gene ontology (GO) Venn diagram of ZoPP2Cs

To better understand the gene functions of *ZoPP2Cs*, we performed gene ontology (GO) annotation analysis using the EggNog website. Based on protein sequence similarity, the 97 *ZoPP2Cs* were classified into 27 functional groups and divided into three categories: biological process (BP), molecular function (MF), and cellular component (CC) (Fig. 8). Within the biological process category, the majority of *ZoPP2Cs* were involved in metabolic process (GO:0008152), cellular process (GO:0009987), and response to stimulus (GO:0050896). In addition, 18 and 2 *ZoPP2C* members were predicted to be involved in plant development (GO:0032502) and immune system response (GO:0002376), respectively. Further, the molecular functions of *ZoPP2Cs* were mainly enriched in catalytic activity (GO:0003824). The cellular components showed that *ZoPP2Cs* worked in cell (GO:0005623), cell part (GO:0044464) and membrane (GO:0016020) (Fig. 8A). The Venn diagram leaded to the conclusion that only 19 genes have single function and 26 genes have both biological processes and molecular functions (Fig. 8B). In order to obtain more information, the GO functions of these 26 annotated to level 2 *ZoPP2C* were analyzed by computational statistics, and the results indicated that *ZoPP2Cs* had transcriptional activity and regulatory function, and played an important role in growth, metabolism and response to stimuli in ginger.

### Expression pattern of *ZoPP2Cs* in different tissues and at different growth stages of ginger

**Fig. 9:**
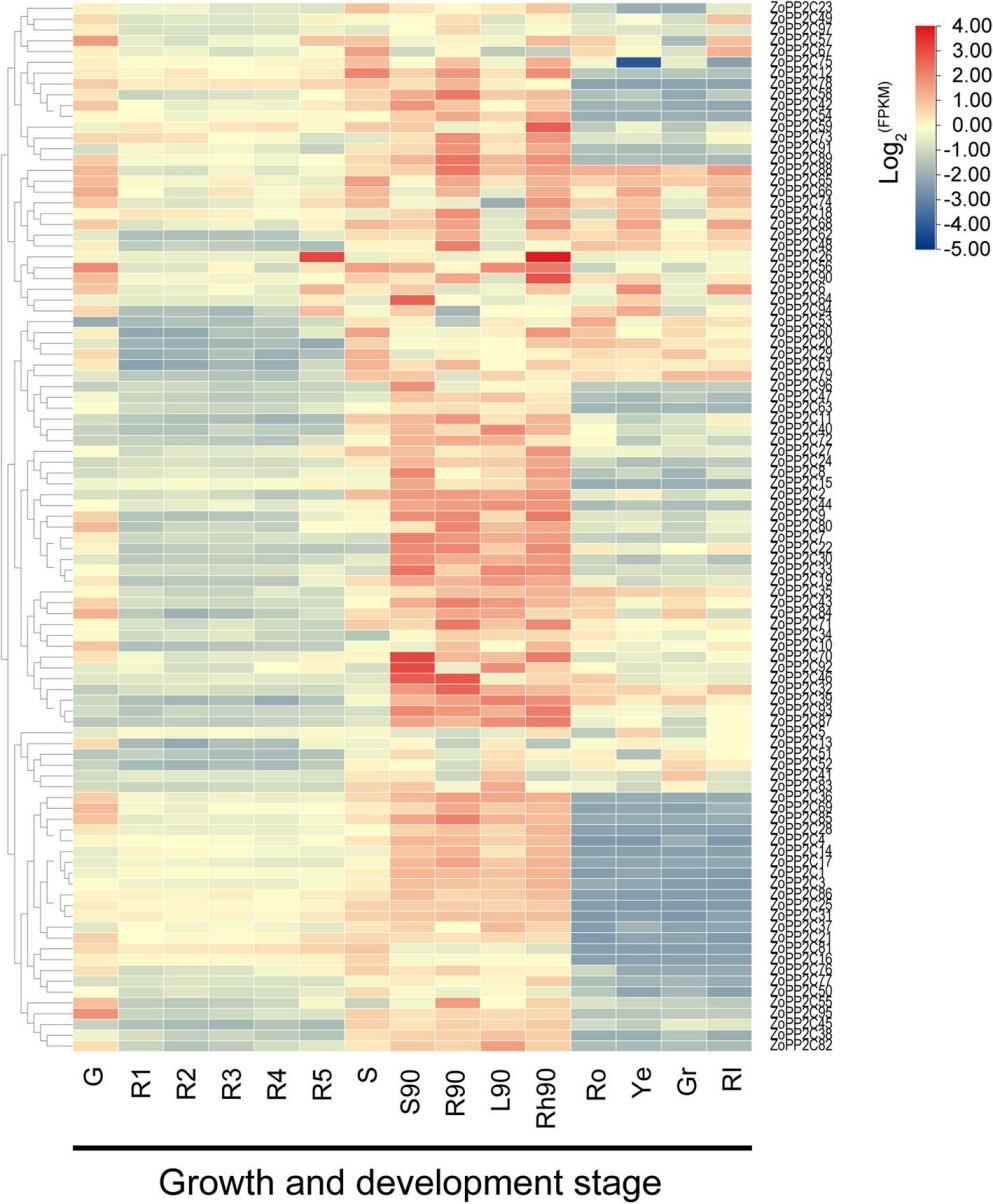
Expression profiles of ZoPP2C gene family. Hierarchical clustering analysis was conducted on the expression profiles of the ZoPP2Cs across 15 ginger samples collected from various tissues and different developmental stages. Normalized values (log2FPKM) were used to represent the expression patterns by color gradient, ranging from blue (negative expression level) to red (positive expression level). Different groups represent G: root, R1-R5: five developmental stages of ginger, S: stem, S90: 90d ginger stem, R90: 90d ginger root, L90: 90d ginger leaf, Rh90: 90d ginger rhizome, RO: red outer epidermis of ginger, RI: red outer epidermal inner tissues of stem, Ye: yellow rhizome, Gr: green rhizome

To investigate the role of *ZoPP2Cs* in different growth and development stages of ginger, we analyzed the expression levels of each *ZoPP2Cs* according to RNA-seq data. Based on the log_2_-transformed FPKM (fragments per kilobase of transcript per million fragments mapped) values of the dataset, the heatmap shows the expression patterns of *ZoPP2Cs* at different growth stages of ginger and under different stress conditions (Table S9). The expression patterns of *ZoPP2C* genes at 90 d in ginger at five developmental stages (R1-R5), root (G), stem (S), as well as leaves (L90), stems (S90), rhizomes (Rh90), and roots (R90) are presented in Fig. 9. All the *ZoPP2Cs* were expressed in roots, stems, and leaves of ginger at different levels. In R1-R5, only *ZoPP2C26* showed high expression level in R5, and the rest of *ZoPP2Cs* showed opposite trends. *ZoPP2Cs* detected in roots (G) and stems (S) including *ZoPP2C95*, *ZoPP2C56*, and *ZoPP2C12*, and *ZoPP2C67* showed higher expression levels. Differently, most *ZoPP2Cs* including *ZoPP2C59*, *ZoPP2C26*, *ZoPP2C90*, *ZoPP2C70*, *ZoPP2C92*, *ZoPP2C46*, *ZoPP2C32* appeared to be expressed in L90, S90, RH90 and R90, with significantly higher expression levels than that in R1-R5, suggesting that the *ZoPP2Cs* would show positive expression trend in the leaves, stems, rhizomes, and root tissues during the maturation stage.

### Expression patterns of the *ZoPP2Cs* under salt, drought, chilling, flooding stresses and *Fusarium solani* infection

**Fig. 10:**
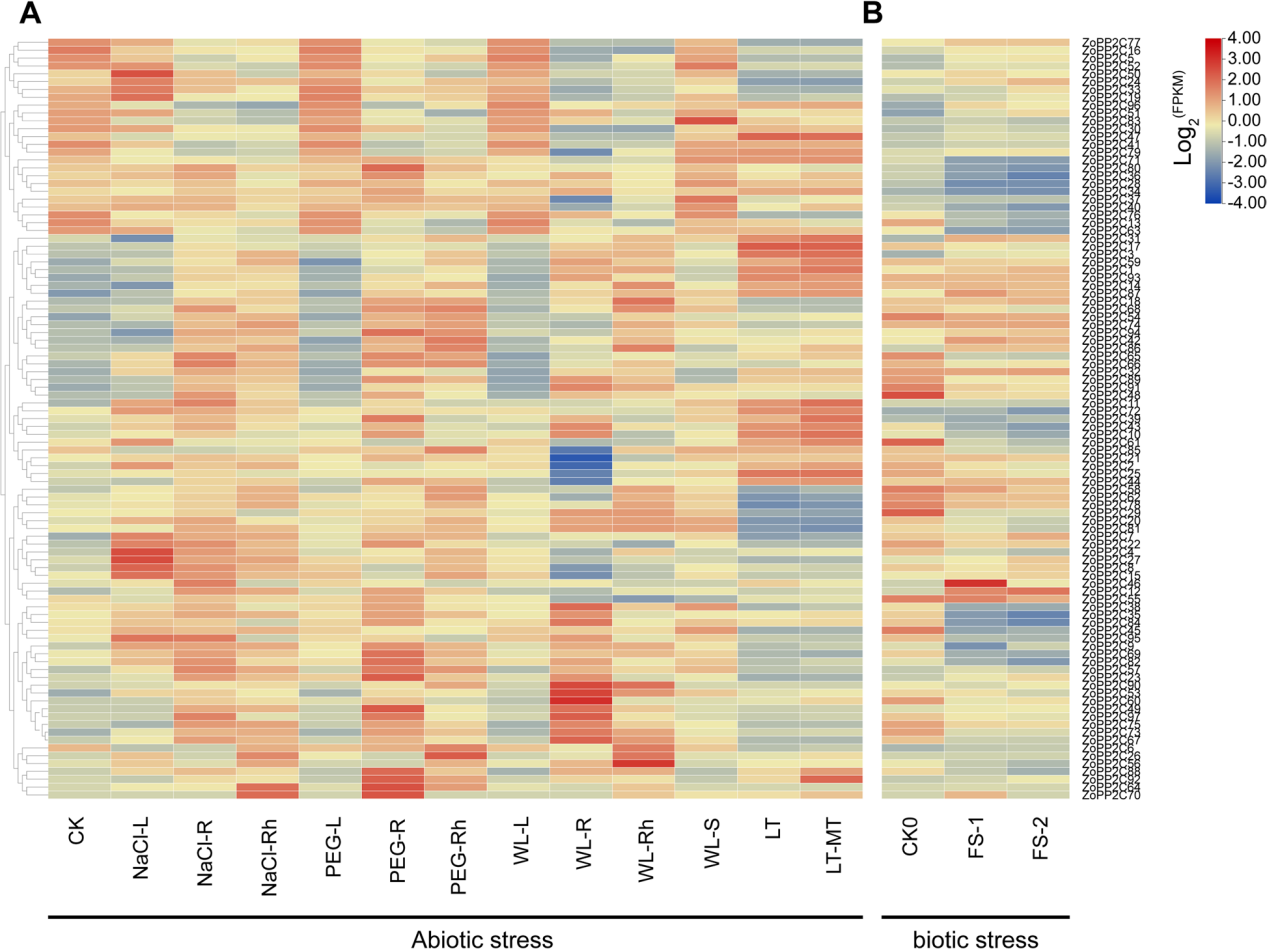
Expression profiles of the ZoPP2Cs under salt, drought, chilling, flooding stresses, and Fusarium solani infection. **A** Heat map of ZoPP2Cs under salt, drought, chilling, flooding stresses. **B** Heat map of ZoPP2Cs after Fusarium solani infection. Expression pattern color bars indicated normalized values (log2FPKM) ranging from blue (negative expression level) to red (positive expression level). The different groups represent NaCl-L: salt stress leaves, NaCl-R: roots, NaCl-Rh: rhizomes; PEG-L: drought stress leaves, PEG-R: roots, PEG-Rh: rhizomes; WL-L: flooding stress leaves, WL-R: roots, WL-Rh. rhizomes, WL-S: stem; LT: chilling stress, LT + MT: chilling stress and exogenous melatonin; CK0: not infested with Fusarium solani, FS-1: 1d after infestation, FS-2: 2d after infestation

According to the analysis of *cis*-elements of *ZoPP2Cs* which contain low-temperature-responsive (LTR), drought-responsive (MBS), and defense-stress-responsive element TC-rich repeats, we analyzed the expression patterns of the *ZoPP2Cs* under salt, drought, cold, and flooding stresses. The results showed most of the *ZoPP2Cs* genes were induced by chilling, drought, or flooding stresses with different expression patterns (Fig. 10A). Under salt stress, the expression levels of most *ZoPP2Cs* including *ZoPP2C50*, *ZoPP2C4*, *ZoPP2C27*, *ZoPP2C8*, and *ZoPP2C88* were significantly increased in leaves (NaCl-L), rhizomes (NaCl-R), and roots (NaCl-Rh) compared with CK. Under drought stress, the expression patterns of most *ZoPP2Cs* showed no significant difference in leaves (PEG-L) compared with CK, and the *ZoPP2Cs* including *ZoPP2C80*, *ZoPP2C94*, *ZoPP2C23*, *ZoPP2C49*, *ZoPP2C26*, *ZoPP2C88*, *ZoPP2C92*, *ZoPP2C64*, and *ZoPP2C70* were elevated in rhizomes (PEG-R) and roots (PEG-Rh). Under flooding stress, the expression patterns of *ZoPP2Cs* in leaves (WL-L1), rhizomes (WL-R1), and roots (WL-Rh1) were similar to that under drought stress. *ZoPP2C90*, *ZoPP2C60*, *ZoPP2C56*, *ZoPP2C85*, *ZoPP2C21*, *ZoPP2C2*, and *ZoPP2C25* were positive expressed in rhizomes (WL-R1), and roots (WL-Rh1) compared to CK, whereas almost unchanged in leaves (WL-L1) and stems (WL-S1). By analyzing the expression profiles of *ZoPP2Cs* genes in leaves of ginger under 25 °C (CK), 2 °C (LT) and 2 °C + melatonin (LT-MT), it was found that some of the *ZoPP2Cs* including *ZoPP2C47*, *ZoPP2C34*, *ZoPP2C17*, *ZoPP2C10* were greatly elevated under LT and LT-MT compared with CK. While some *ZoPP2Cs* like *ZoPP2C78* and *ZoPP2C20* were negatively expressed under chilling stress, and their expression levels were significantly decreased under LT-MT. In addition, after *Fusarium solani* infestation, the expression levels of most of the *ZoPP2Cs* were gradually decreased along with the time of infestation (Fig. 10B). The results revealed that the majority of *ZoPP2Cs* were significantly expressed under abiotic stresses, albeit with varying expression patterns. Furthermore, the expression of *ZoPP2Cs* was notably up-regulated in ginger rhizomes in comparison to leaves.

### RT-qPCR analysis

**Fig. 11:**
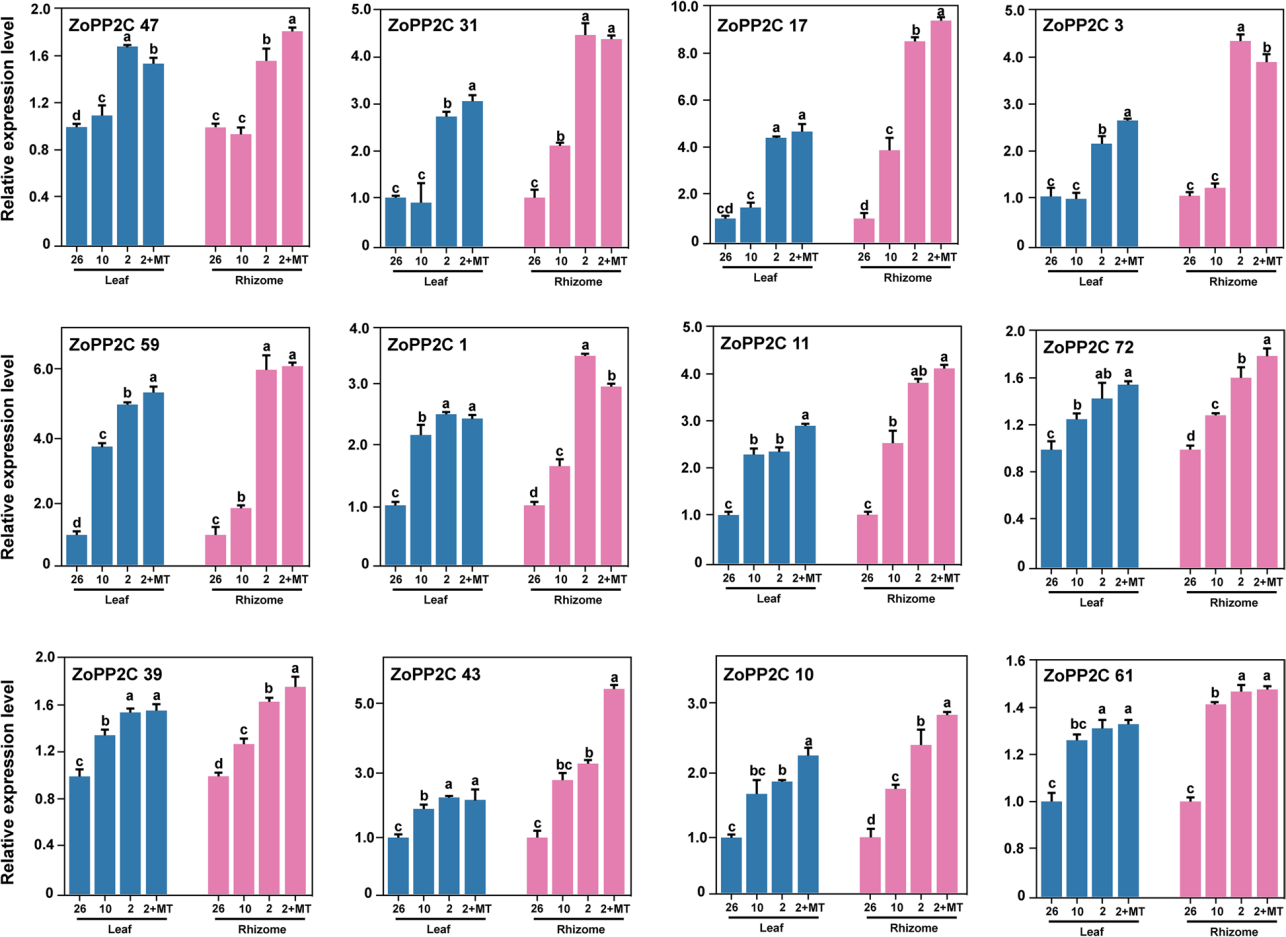
Relative expression levels of 12 selected ZoPP2C genes under chilling treatment by qRT-PCR. Blue color represents the gene expression profile in ginger leaves. Red color represents the expression profile in ginger rhizomes. The four treatments were 26°C, 10°C, 2°C, and 2°C + melatonin (LT + MT). Error bars represent the standard deviation of three independent biological replicates

To further understand the expression pattern of *ZoPP2C* genes in chilling stress and to check the reliability of the existing transcriptome FPKM, we selected 12 *ZoPP2C* genes that were significantly up-regulated by chilling stress based on the heat map, and analyzed the expression level of each *ZoPP2C* gene in different tissues, including mature leaves and rhizomes using RT-qRCR. The results showed (Fig. 11) that the expression levels of the 12 selected *ZoPP2Cs* were generally higher in rhizomes than in leaves. Under chilling stress (2 °C), *ZoPP2C31*, *ZoPP2C17*, *ZoPP2C3*, *ZoPP2C59*, *ZoPP2C1*, *ZoPP2C11*, and *ZoPP2C43* were significantly up-regulated in both leaves and rhizomes, among which *ZoPP2C31* was up-regulated 2.8- and 4.4-fold in leaves and rhizomes under chilling stress compared with CK, respectively. *ZoPP2C17* was up-regulated 4.1- and 8.2-fold in leaves and rhizomes, respectively. *ZoPP2C59* was up-regulated 4.9- and 5.8-fold in leaves and rhizomes, respectively. *ZoPP2C11* was up-regulated 2.2- and 3.9-fold in leaves and rhizomes, respectively. In addition, the expression patterns of *ZoPP2C17*, *ZoPP2C59*, *ZoPP2C11*, *ZoPP2C72*, *ZoPP2C43*, and *ZoPP2C10* in ginger leaves and rhizomes were significantly increased under chilling stress after melatonin treatment (LT + MT), among which the expression level of *ZoPP2C17* in leaves and rhizomes after adding MT treatment at 2 °C increased 0.6-fold and onefold compared with 2 °C, respectively, and *ZoPP2C43* in rhizomes increased 2.2-fold compared with that under LT + MT conditions. The results showed that the expression of some *ZoPP2Cs* under chilling stress would also be changed by exogenous substances.

## Discussion

Environmental factors strongly influence the distribution and growth of ginger [28]. Therefore, developing highly resistant ginger varieties that can withstand various adversities is a crucial aspect of the advancement for ginger cultivation industry. Genetic engineering is essential in enhancing vegetable varieties beyond the confines of traditional breeding techniques [30], and identifying genes with regulatory functions against adversities is now crucial for promoting genetic breeding through genetic engineering [31, 32]. Additionally, PP2C has been found to function in plant growth and development, along with protecting against biotic and abiotic stresses [9, 33, 34]. Therefore, it is imperative to conduct a comprehensive investigation of PP2C and uncover its response to abiotic stress mechanisms in ginger. The recent achievement of high-quality ginger genome sequencing data [35] provides a crucial reference for exploring the functional characteristics of ginger genes, and this breakthrough facilitates the precise identification of PP2C gene family in ginger.

Compared to *A. thaliana* (94), banana (87) [36], cucumber (56) [37], cotton (87) [14] and mulberry (63) [38], 97 *PP2C* genes were identified from the ginger genome, which suggesting the considerable variability of *PP2C* gene families between different species. The physicochemical property investigation ascertained the ZoPP2C proteins predominately localize to the nucleus, that is comparable to PP2Cs in other species. For example, *A. thaliana* PP2C members RDO5 and DBP1 [6], as well as the rice OsSIPP2C1, are located in the nucleus [20]. Whilie in tomato, some PP2Cs are reported to located in the cytoplasm [39]. In rice, the OsPP108 is expressed both in the cytoplasm and nucleus. Previous research has highlighted distinctive structural domains and motifs in plant PP2C proteins, indicating that structural diversity could be a crucial factor in their functional diversity [40]. In this study, we evaluated the conserved structural domains of ZoPP2Cs through multiple sequence comparisons and found that some ZoPP2C amino acid sequences were mutated, whereas the PP2C protein sequences were more conserved in *Arabidopsis thaliana*, which may lead to a series of changes in the protein structure of ZoPP2Cs. Furthermore, in eukaryotes, the *PP2C* gene family exhibits a prevalent unified structural attribute comprising an operative catalytic structural domain and incorporating 11 conserved motifs, typically situated in either N-terminal or C-terminal positions [7]. In *A. thaliana*, the catalytic structural domain is generally situated at the C-terminus, while the N-terminus contains a variable-length and weakly conserved extended region. These different extension lengths have an influence on the PP2C function [41]. In contrast, ZoPP2Cs in ginger have extended regions of varying length at both the N- and C-terminus, which means that ZoPP2Cs may have more diverse biological functions. What’s more, the 97 *ZoPP2C* genes were phylogenetically classified into 15 subclades (A-O) [9] based on the classification of the *A. thaliana AtPP2Cs* which have been divided into 10 subclades (A-J). Genes in the same subfamily may possess comparable biological characteristics. Further evidence for the phylogenetic relationships can be acquired through the investigation of conserved motifs and gene structures. The subfamilies of ZoPP2Cs have comparable motif patterns and domains. This discovery is backed by the research on PP2C genes in *A. thaliana* [9, 13], soybean [11], and cucumber [37]. The PP2Cs in the subfamily also possess structural specificity that could explain the dissimilar functions of the subfamilies [21]. It is noteworthy to mention that ZoPP2C variations in exon–intron number and arrangement are extensive. There are also significant differences in these variants between *ZoPP2C* subfamilies, such as molecular weight, pI value, and uneven chromosome distribution. Shazadee et al. [14] investigated that the cotton GaPP2Cs display significant differences in protein length, molecular weight, isoelectric point, and hydrophilicity averages. The results suggest that PP2C proteins from distinct subfamilies may have complex functions in diverse microenvironments. *PP2C* genes of the same subfamily share the same exon/intron structure. However, large quantitative and structural differences were observed between the different subfamilies, forming a pattern that may be closely related to the catalytic core domain of PP2C proteins [4, 13]. The present study is in support of these findings.

Gene duplication, including fragment and tandem duplication, is widely acknowledged as a major driving force in genome evolution [42]. Replication of chromosome fragments or entire genomes is a major source of evolution, including the generation of new genes with altered functions and expression patterns [43]. Our study found that PP2Cs in *A. thaliana* [13] and soybean [44] lack tandem duplication events, which could be attributed to genomic segregation of transposon activity and the relocation and duplication of individual genes [45]. Collinearity analysis demonstrated that the amplification of ZoPP2Cs predominantly proceed through fragment duplication. This method is more advantageous for maintaining gene function during duplication than tandem duplication [46, 47]. For example, ZoPP2C87 and ZoPP2C93 are a pair of duplicated genes, both of which have similar expression patterns at the developmental stage of ginger, as compared to under stress treatments. However, this study has also found that the duplicate gene pairs, ZoPP2C2 and ZoPP2C96, are expressed differently in the same parts of ginger. Subsequent studies found that the motif positions and composition of these genes were identical, suggesting that the differential expression of the two may be attributed to gene mutations that occur during gene duplication, resulting in the loss of certain gene fragments. The Ka/Ks values demonstrate the pressure of selection on gene pairs throughout the course of evolution. In *ZoPP2Cs*, the Ka/Ks values of 33 pairs of covariates are less than 1 and are in varying proportions, indicating that the *ZoPP2C* homologous gene pairs might have undergone different levels of purifying selection during evolution process to reduce the incidence of deleterious mutations in the population. However, duplicated genes can be functionally redundant [32], and investigating the *ZoPP2Cs* functionality would yield useful insights into their potential role in future evolution. In addition, cross-species collinearity analyses showed that *ZoPP2C64* and *ZoPP2C65* were present in PP2C homologous gene pairs in alfalfa, cucumber, soybean, and banana, and that these two *ZoPP2Cs* may play an important role in species genetic differentiation. Banana and ginger have the highest degree of collinearity. Both banana and ginger belong to the same monocotyledonous tropical ginger genus and have evolved from a common ancestor [48, 49]. Consequently, some of their *PP2C* genes may have similar structural and functional characteristics. Due to the limitations of current genetic transformation techniques in ginger, it is more advantageous to use kindred plants to assess the function of *PP2C* genes than in model plants such as *A. thaliana*. This area will be the main focus of *PP2Cs* research in the future.

*Cis*-elements located in the promoter regions are closely associated with gene transcription, and they have significant functions in plant signal transduction by interacting with transcription factors [50]. Several *cis*-regulatory elements related to phytohormones, plant growth and development, biotic and abiotic stresses were identifid in the promoter regions of *ZoPP2Cs*. Some of these *cis*-elements have been previously observed in the research ostudies of cotton [14], cucumber [37], and strawberry [51]. The identification of *cis*-regulatory elements in *ZoPP2C* suggests that these genes may play crucial function in responding to diverse stresses and regulating phytohormones. Most of the *cis*-regulatory elements including ABRE, CGTCA-motif, and TGACG-motif in *ZoPP2Cs* are related to phytohormones. ABRE mainly works in responding to ABA and contributing to regulate the plant tolerance to drought, high temperature, and chilling stresses [52]. CGTCA and TGACG motifs act as JA response elements [53, 54] and may have an effect on the transcription level and stress response of *ZoPP2Cs* under different adverse conditions. It is noteworthy that the presence and quantity of these response elements may be the key for plants to adapt to environmental stresses.

In-depth investigation of plant protein–protein interaction networks (PPI) is vital to understand the fundamental working mechanism of proteins, their responses to different stress signals and material metabolism, as well as their interconnections [55]. In this study, 5 hub proteins were selected by PPI and categorized into three subfamilies. ZoPP2C9, ZoPP2C23, and ZoPP2C26 were grouped into subfamily K, ZoPP2C49 and ZoPP2C92 belonged to subfamily A and J, respectively. PP2C can regulate the MAPK signaling pathway and participate in the regulation of stress-related signaling pathways by directly or indirectly interacting with certain components of the MAPK cascade response [7, 18]. In maize, ZmPP84 has been shown to negatively regulate stomatal closure and drought stress by dephosphorylating ZmMEK1 and inhibiting the ZmMEK1-ZmSIMK1 signaling pathway [56]. KEGG enrichment analysis suggests that the genes including ZoPP2C9, ZoPP2C23, ZoPP2C26, ZoPP2C49 and ZoPP2C92 may have important functions in regulating MAPK signaling pathway especially in the process of stress response, but further research is needed to clarify ZoPP2C genes' unique specificity in stress response and its role in improving ginger genetics.

TFs often regulate multiple target proteins simultaneously, and their binding sites show a certain degree of conservation but not identical [57]. This study demonstrates that ZoPP2Cs could be regulated by several TFs, which results in more diverse functions of ZoPP2Cs, such as regulating abiotic stress responses, plant growth and development. AP2/ERF transcription factors are involved in various environmental stress response processes, including cold, heat, drought, and salinity [58, 59]. Research indicates that the *ZoAP2* genes have a significant impact on ginger growth and development [28]. While, *ZoPP2Cs* were found to have different AP2/ERF binding sites, which may have a combined function in regulating both adversity and growth/development, but further investigation is needed.

PP2C members are essential in the process of plant organogenesis and fruit ripening. For example, the *AtPP2CF1* is proved to be involved in promoting root tip and leaf growth, stimulating stem development and increasing biomass in *A. thaliana* [9]. Similarly, the tomato *SlPP2C1* has been found to accumulate in the pericarp, seed and pulp of fruit, and also function in accelerating fruit ripening [39]. In this study, *ZoPP2Cs* were identified in the leaves, stems, and rhizomes of ginger, and their expression levels varied slightly during different growth stages. Notably, the majority of *ZoPP2Cs* were significantly up-regulated during the late growth stage of ginger (90 d), suggesting that they may have potential functions at this developmental stage and play an important role in ginger ripening. Additionally, *ZmPP2C42* and *ZmPP2C47* were found to have the highest expression in mature pollen in maize, indicating their potential role in maize maturation [4, 60]. Ginger yield and quality are directly affected by efficient development and expansion of rhizomes, highlighting their importance in production. According to the transcriptomic data available, *ZoPP2C88*, *ZoPP2C89*, *ZoPP2C46*, and *ZoPP2C32* were up-regulated significantly in rhizomes. It is hypothesized that these *ZoPP2Cs* might participate in the expansion process of ginger rhizomes.

Numerous studies have confirmed the involvement of several *PP2Cs* in regulating various plant response mechanisms to stresses, particularly chilling [12, 61]. Prior research has reported that postharvest ginger was exposed to chilling stress at 2°C, resulting in a surge of ABA and down-regulation of the expression of some *PP2C* genes [27]. In this study, it was discovered that certain *ZoPP2Cs* were considerably expressed in instances of chilling stress in ginger using RNA-seq and qRT-PCR. It was observed that *ZoPP2Cs*’ expression levels were higher in rhizomes compared with leaves. It was established that a vast number of *PP2C* genes were significantly expressed in sugarcane under chilling stress, and still experienced an increase in expression alongside the duration of stress [61]. Some of the *PP2C* genes in *A. thaliana* like *AP2C1* were highly induced by drought and chilling, with notable differences in the expression level across various tissues [9, 21]. In addition, during chilling stress, the majority of *ZoPP2Cs* belong to J are likely to be critically involved in this type of stress. It has been revealed the expression levels of *PP2C* subfamily A in *A. thaliana* and rice showed significant changes under chilling stress, and they were also discovered as negative regulators of ABA-mediated signaling pathways [62, 63]. It is widely held that introducing exogenous substances like ABA can regulate plant cold tolerance due to the concurrent activation of ABA signaling during plant chilling stress responses [52, 64]. For example, some of the *FvPP2Cs* exhibited significant elevation in woodland strawberry under chilling stress with the addition of ABA [51]. It was hypothesized that these genes functioned as positive regulators in the ABA signaling response. In this study, we found a significant increase in the expression levels of certain *ZoPP2Cs* under chilling stress following application of external melatonin in ginger, suggesting that *ZoPP2Cs* may contribute to improve the MT-induced chilling resistance.

In addition, it is noteworthy that certain *ZoPP2Cs* were significantly down-regulated under chilling stress. The model of the PYL (PYR/PYL/RCAR)-PP2C-SnRK2 ABA signaling pathway emerged as the most important network in response to chilling stress [65, 66]. Studies have demonstrated that PP2C interacts with SnRK2 to inhibit cold tolerance in plants [11, 67]. The rice *OsPP2C27* can negatively regulate chilling tolerance in plants [20, 62]. Therefore, the significant down-regulation of *ZoPP2Cs* in this study implies that these genes may act as negative regulators in chilling stress. In addition, *ZoPP2Cs* exhibited different expression levels under various stress conditions, suggesting that *PP2C* genes execute multiple functions depending on the type of stress encountered. All the results showed that using phenotypic data to screen *ZoPP2Cs* response to abiotic and biotic stresses is contributing to screening out genotypes and varieties with higher resistance, and providing an important theoretical basis for the future breeding of highly resistant varieties.

## Conclusions

In this study, a total of 97 *ZoPP2Cs* were identified in ginger and classified into 15 classes. The results indicated that *ZoPP2Cs* were evolutionarily related to banana *PP2C* genes, perhaps originated from the same ancestor. All *ZoPP2Cs* were amplified through fragment duplication. ZoPP2Cs proteins predominantly functioned in the nucleus (68) and chloroplasts (20). Additionally, ZoPP2Cs of the same subfamily displayed comparable motif patterns and exon/intron arrangement structures. Analysis of *cis*-acting elements in the promoter region revealed that the *ZoPP2C* genes primarily respond to hormonal and abiotic stresses and regulate the ginger development. According to the PPI network, ZoPP2C9, ZoPP2C23, ZoPP2C26, ZoPP2C49, and ZoPP2C92 proteins had significant interactions and functioned as potential hub proteins. This study also investigated the expression patterns of *ZoPP2Cs* in response to different abiotic stresses using RNA-seq and qRT-PCR analyses, and found that *ZoPP2Cs* were involved in coping with salt, drought, flood and chilling stresses. Several *ZoPP2Cs* including *ZoPP2C17*, *ZoPP2C59*, *ZoPP2C11*, *ZoPP2C72*, *ZoPP2C43* and *ZoPP2C10* showed significant up-regulation patterns in response to chilling, which may represent a crucial adaptive response. To conclude, these lines of evidence may provide valuable insights into the molecular mechanisms of *ZoPP2Cs* in response to abiotic stresses, specifically the chilling stress.

## Materials and methods

### Plant materials

The ginger of ‘Shandongdajiang’ was utilized in this study. Germination occurred in an incubator under controlled environmental conditions of 25 °C temperature, 65% relative humidity, and a 24 h photoperiod of darkness. Once the buds reached 1.5 cm in length, healthy ginger seedlings were selected and planted in a glass greenhouse. To assess the expression levels of *ZoPP2Cs* in different ginger tissues, we collected ginger leaves, stems, rhizomes and roots at five growth stages and at 90 d of growth. To examine the role of *ZoPP2Cs* during distinct abiotic stresses, two-month-old ginger seedlings with five leaves were utilized. For the salt treatment, the ginger seedlings were subjected to root irrigation using a NaCl solution with a concentration of 20 gL^−1^ for 3 d. Under drought treatment, the ginger seedlings were treated with PEG-6000 at 25% for 7 d. Under flooding treatment, ginger seedlings were placed in a water tank (specifications: 150 cm in length, 120 cm in width, and 50 cm in height) where the water surface was always 3 cm above the substrate soil in the pots for 3 d; Under the chilling treatment, the ginger seedlings were divided into two groups. One group was placed at 2 °C, while the other group was placed at the same temperature and sprayed with melatonin (MT) at the same time. The co-treatment continued for 3 d. Furthermore, a control group was established and the ginger seedlings were maintained as usual. There were 9 pots of ginger seedlings in each treatment group. Ginger leaves, rhizomes and roots were harvested at each time point under each treatment. Samples were rapidly frozen in liquid nitrogen and preserved at -80 °C for subsequent analysis.

### Identification of *ZoPP2C* genes in ginger

To comprehensively characterize the *ZoPP2C* genes in ginger, we followed these steps: Step 1, we obtained the Hidden Markov Model (HMM) of the PP2C structural domain (PF00481) from the Pfam Protein Family Database (http://pfam.xfam.org/) with an e-value of 1 × 10^–5^; Step 2, The amino acid sequences of AtPP2Cs were obtained from The *A. thaliana* Information Resource (TAIR) (http://www.arabidopsis.org/index.jsp) and the ZoPP2Cs were from the whole ginger genome, respectively; Step 3, Validate the accuracy of the inferred protein sequences through a search for homologous sequences in the GenBank database (non-redundant) by utilizing the BLASTp tool (Basic Local Alignment Search tool for proteins) with an e-value of 1 × 10^–5^; Step 4, Verify the accuracy of the deduced protein sequences by searching for comparable sequences in the non-redundant GenBank database using the NCBI conserved domain database (https:/ /www.ncbi.nlm.nih.gov/cdd/) (e-value of 1 × 10^–5^ and default parameter settings) and the SMART database (http://smart.embl-heidelberg.de/) to select potential protein sequences. Assess the presence of PP2C structural domains and manually remove the structural domains of the nonconforming *PP2C* gene.

### Characterization of ZoPP2C proteins in ginger

The Compute pI/MW tool (http://web.expasy.org/Compute) from the ExPASy server was used to determine various protein characteristics of the ZoPP2C proteins including amino acid length (AA), molecular mass (MW), isoelectric point (PI), hydrophilicity (dGRAVY), and instability index. The online prediction tool BUSCA (http://busca.biocomp.unibo.it/) was used to predict the subcellular localization of the ginger PP2C proteins.

### Multiple sequence alignment, tertiary structure, phylogenetic tree and chromosome localization

The Interpro database (https://www.ebi.ac.uk/interpro/) was used to query the conserved domains of PP2C proteins, whilst Jalview software (v2.11.2.0) was employed to compare and edit them and then generate the conserved motif logos. The AlphaFold protein structure database (https://alphafold.ebi.ac.uk/) was used to obtain the tertiary structure of the PP2C structural domain. Per-residue confidence scores (pLDDT) from AlphaFold2 range from 0 to 100. The pLDDT below 50 may have lower confidence levels.

Homology analysis of ZoPP2Cs was conducted based on a previous study [37]. PP2C protein sequences of *A. thaliana* was obtained from the TAIR and NCBI databases. The Clustal W program was used to perform multiple sequence comparisons between *A. thaliana AtPP2C* and ginger *ZoPP2Cs*. Subsequently, phylogenetic analyses were carried out using MEGA X [68], utilizing the multiple sequence comparisons from partial deletion, 80% truncation, JTT + G + I + F amino acid substitution model, and 1000 bootstrap repetitions of the MEGA X program to construct the ML phylogenetic tree. The ML tree was obtained and subsequently uploaded to iTOL (https://itol.embl.de/) [28] for generating the dendrogram. To determine the location of ZoPP2Cs on the ginger chromosomes, the *ZoPP2C* genes’ physical position on the ginger chromosome was extracted using TBtools from the ginger genome GFF file and then structured into a chromosome density file.

### Gene structure, conserved protein motifs and exon–intron structure

The structural domains of ZoPP2C proteins were validated through the online tool (https://www.ncbi.nlm.nih.gov/Structure/cdd/wrpsb.cgi). To identify conserved motifs within ZoPP2C proteins, we used the online program Multiple Em for Motif Elicitation (MEME v5.5.0) (https://meme-suite.org/meme/) with the zoops option for site distribution selection. All parameters were set to their default values. The number of repetitions was determined arbitrarily, with a maximum of 20 patterns considered. The distribution of introns and exons in ZoPP2Cs was acquired through the GFF annotation file from the ginger genome.

### Collinearity and gene duplication analysis

The location of the *ZoPP2Cs* was extracted from the gff3 genome annotation file using TBtools (v1. 123), and gene duplication events were analyzed with the Multiple Covariance Scanning Toolkit (MCScanX). To demonstrate the homozygous PP2C gene identity obtained from ginger and other species, (*A. thaliana*, cotton, alfalfa, cucumber, soybean, and banana), the collinearity relationship was analyzed. The NG model of KaKs_Calculator 3.0 software was used to calculate the nonsynonymous substitution rate (Ka), synonymo us substitution rate (Ks), and Ka/Ks values for all duplicate gene pairs. The differentiation time of the *ZoPP2Cs* (MYA: millions of years ago) was determined using the reference equation: T = Ks/2λ (where λ = 6.5 × 10^–9^) [32].

### *Cis*-acting element analysis of the ZoPP2C promoters

To identify the *cis*-acting elements in the promoter sequences of ginger *ZoPP2C* genes, the upstream 2,000 bp sequence of *ZoPP2Cs* was scrutinized through the PlantCARE online database (https://bioinformatics.psb.ugent.be/webtools/plantcare/html/). Afterwards, the *cis*-acting elements were categorized and tallied, and demonstrated in histograms using sigma plot (v12.5) software. Additionally, heatmaps displaying various categories of *cis*-acting elements were generated by the Heatmap R package.

### Protein–protein interaction and TF binding site network analysis

Interactions among ginger ZoPP2C proteins were predicted through the online interacting protein search site STRING (http://string-db.org/). PlantRegMap (http://plantregmap.gao-lab.org/) was then used to analyze all transcription factors that might potentially bind to the upstream region (2,000 bp) of ZoPP2Cs, and Cytoscape (v3.9.1) (http://www.cytoscape.org/) was used to map and enhance protein–protein interaction networks as well as transcription factor interactions with ZoPP2Cs. Moreover, the ggplot2 package in R (Ginestet, 2011was used to generate word clouds.

### GO functional annotation and enrichment analysis of ZoPP2Cs

The transcriptional functions of *ZoPP2Cs* were annotated using Blast2go software (https://www.blast2go.com/free-b2gtrial). The annotation results were further analyzed using GO function annotation. GO enrichment heatmap was plotted using R package Heatmap.

### Using RNA-seq and qRT-PCR analysis to determine the expression of *ZoPP2Cs*

Transcriptomic data was obtained from various growth stages of ginger, as well as from ginger exposed to salt, drought, flooding, chilling stresses, and *Fusarium roqueforti* infestation. We employed a strict algorithm to identify genes that were differentially expressed in the ginger defense response. The false discovery rate (FDR) was established at 5%, while the *P*-value threshold for multiple comparative tests and analyses were determined by manipulating the FDR value. Thresholds for determining the significance of gene expression differences were set at *P* < 0.001 and log2 ratio absolute value > 1. *ZoPP2Cs* gene expression levels were calculated through Cufflinks (FPKM values, Fragments per kilobase of exon 150 per million reads mapped), and TBtools' Heatmap function was used to produce heat maps [69]. Primers for qRT-PCR were designed via Primer 5 software (Sangon Biotech (Shanghai) Co., Ltd). RNA was extracted using the Trizol method and RNA quality was assessed via 1% agarose gel electrophoresis and DL2000x Maker to ensure the integrity of the total RNA. The first strand cDNA was synthesized by following the reverse transcription kit (Vazyme Biotech Co., Ltd) and stored at -20°C for future use. SYBRqPCR MasterMix (Vazyme Biotech Co., Ltd) was used for qRT-PCR amplification [70], and the RBP gene used as an internal reference. The amplification reaction mix (20 μL) contained 10 μL of SYBRPremix, 1 μL each of 169 (10 μmol/L) upstream and downstream primers, 2.5 μL of cDNA, and the rest was made up of ddH_2_O. Reaction procedures: Prior to denaturation, samples were incubated at 95°C for 3 min. Denaturation lasted for 15 s at 95°C, followed by annealing at 60°C for another 15 s and then at 65°C for 5 s. A total of 40 cycles of denaturation and annealing were performed. The experiment was conducted with three biological replicates for each sample, and gene expression levels were determined using the 2^−ΔΔCt^ method.

### Statistical analysis

Analysis of variance (ANOVA) was performed using IMB SPSS statistics, Duncan's test was used for significance calculation (*P* < 0.05), and GraphPad Prism 9 was used for plotting histograms.

### Supplementary Information

**Additional file 1: Table S1.** ZoPP2C gene correspondence. **Table S2.** Identification of PP2C genes in ginger (ZoPP2C). **Table S3.** Segmental replication covariate gene pair Ka/Ks analysis of ZoPP2Cs. **Table S4.** Duplicate gene pairs between different species. **Table S5.** ZoPP2C protein interaction network connection degree. **Table S6.** ZoPP2Cs Protein Interaction Network KEGG Enrichment Analysis. **Table S7.** ZoPP2C and potentially binding transcription factors (TFs). **Table S8.** The number of transcription factor binding sites. **Table S9.** Source of data sets of transcriptomes of ginger.

**Additional file 2:** **Fig. S1.** ZoPP2C gene chromosomal localization. **Fig. S2.** 1-20 motif sequence logos.

## Data Availability

No datasets were generated or analysed during the current study.
