# Supplementary material for: Genome-wide analysis and expression pattern of the ZoPP2C gene family in Zingiber officinale Roscoe
Source: BMC Genomics. 2024 Jan 20;25:83. doi: 10.1186/s12864-024-09966-w (PMC10799369; doi:10.1186/s12864-024-09966-w)
Supplement: Supplementary file 1 — Additional file 1: Table S1. ZoPP2C gene correspondence. Table S2. Identification of PP2C genes in ginger (ZoPP2C). Table S3. Segmental replication covariate gene pair Ka/Ks analysis of ZoPP2Cs. Table S4. Duplicate gene pairs between different species. Table S5. ZoPP2C protein interaction network connection degree. Table S6. ZoPP2Cs Protein Interaction Network KEGG Enrichment Analysis. Table S7. ZoPP2C and potentially binding transcription factors (TFs). Table S8. The number of transcription factor binding sites. Table S9. Source of data sets of transcriptomes of ginger. [file 12864_2024_9966_MOESM1_ESM.docx]

Table S1 ZoPP2C gene correspondence

| Gene ID | Gene NO. |
| --- | --- |
| Maker00040826 | ZoPP2C37 |
| Maker00045279 | ZoPP2C40 |
| Maker00057658 | ZoPP2C38 |
| Maker00011438 | ZoPP2C11 |
| Maker00077313 | ZoPP2C21 |
| Maker00008514 | ZoPP2C39 |
| Maker00076053 | ZoPP2C18 |
| Maker00020808 | ZoPP2C17 |
| Maker00023399 | ZoPP2C14 |
| Maker00018941 | ZoPP2C45 |
| Maker00078600 | ZoPP2C51 |
| Maker00033227 | ZoPP2C28 |
| Maker00050685 | ZoPP2C36 |
| Maker00077383 | ZoPP2C26 |
| Maker00012311 | ZoPP2C13 |
| Maker00054423 | ZoPP2C42 |
| Maker00008359 | ZoPP2C47 |
| Maker00050720 | ZoPP2C91 |
| Maker00004715 | ZoPP2C1 |
| Maker00003607 | ZoPP2C73 |
| Maker00022445 | ZoPP2C96 |
| Maker00035697 | ZoPP2C57 |
| Maker00025175 | ZoPP2C59 |
| Maker00077678 | ZoPP2C77 |
| Maker00023416 | ZoPP2C9 |
| Maker00012737 | ZoPP2C54 |
| Maker00018982 | ZoPP2C2 |
| Maker00077518 | ZoPP2C3 |
| Maker00054432 | ZoPP2C75 |
| Maker00031638 | ZoPP2C69 |
| Maker00000847 | ZoPP2C35 |
| Maker00069164 | ZoPP2C4 |
| Maker00015020 | ZoPP2C60 |
| Maker00045224 | ZoPP2C8 |
| Maker00009631 | ZoPP2C6 |
| Maker00077547 | ZoPP2C72 |
| Maker00046885 | ZoPP2C15 |
| Maker00038450 | ZoPP2C71 |
| Maker00038419 | ZoPP2C5 |
| Maker00034385 | ZoPP2C7 |
| Maker00053921 | ZoPP2C43 |
| Maker00023410 | ZoPP2C41 |
| Maker00001296 | ZoPP2C67 |
| Maker00054137 | ZoPP2C58 |
| Maker00005941 | ZoPP2C34 |
| Maker00023108 | ZoPP2C62 |
| Maker00011427 | ZoPP2C20 |
| Maker00077912 | ZoPP2C63 |
| Maker00058110 | ZoPP2C64 |
| Maker00016824 | ZoPP2C29 |
| Maker00077387 | ZoPP2C80 |
| Maker00000290 | ZoPP2C52 |
| Maker00067319 | ZoPP2C10 |
| Maker00036903 | ZoPP2C70 |
| Maker00078292 | ZoPP2C32 |
| Maker00047171 | ZoPP2C56 |
| Maker00033633 | ZoPP2C78 |
| Maker00028292 | ZoPP2C50 |
| Maker00051403 | ZoPP2C12 |
| Maker00016649 | ZoPP2C90 |
| Maker00069785 | ZoPP2C55 |
| Maker00075838 | ZoPP2C86 |
| Maker00055638 | ZoPP2C22 |
| Maker00078283 | ZoPP2C88 |
| Maker00016218 | ZoPP2C92 |
| Maker00023137 | ZoPP2C23 |
| Maker00021600 | ZoPP2C25 |
| Maker00077694 | ZoPP2C44 |
| Maker00002553 | ZoPP2C27 |
| Maker00033289 | ZoPP2C33 |
| Maker00008990 | ZoPP2C81 |
| Maker00069378 | ZoPP2C74 |
| Maker00027000 | ZoPP2C48 |
| Maker00031948 | ZoPP2C68 |
| Maker00042845 | ZoPP2C65 |
| Maker00044936 | ZoPP2C66 |
| Maker00052338 | ZoPP2C61 |
| Maker00012814 | ZoPP2C46 |
| Maker00021254 | ZoPP2C85 |
| Maker00023265 | ZoPP2C49 |
| Maker00026965 | ZoPP2C53 |
| Maker00052394 | ZoPP2C19 |
| Maker00036709 | ZoPP2C24 |
| Maker00002571 | ZoPP2C89 |
| Maker00012949 | ZoPP2C76 |
| Maker00001113 | ZoPP2C97 |
| Maker00055719 | ZoPP2C95 |
| Maker00034579 | ZoPP2C16 |
| Maker00069522 | ZoPP2C83 |
| Maker00008258 | ZoPP2C31 |
| Maker00000420 | ZoPP2C82 |
| Maker00039734 | ZoPP2C93 |
| Maker00029484 | ZoPP2C87 |
| Maker00016078 | ZoPP2C84 |
| Maker00000554 | ZoPP2C30 |
| Maker00063717 | ZoPP2C94 |
| Maker00025211 | ZoPP2C79 |

Table S2 Identification of PP2C genes in ginger (ZoPP2C)

| Gene NO. | aLength(AA) | MW(Da) | | cpl | dGRAVY | Instability index | | fLoc |
| --- | --- | --- | --- | --- | --- | --- | --- | --- |
| ZoPP2C37 | 273 | | 30185.22 | 6.46 | -0.32 | 43.44 | nucleus | |
| ZoPP2C40 | 273 | | 30183.2 | 6.26 | -0.313 | 43.99 | nucleus | |
| ZoPP2C38 | 284 | | 31205.49 | 8.21 | -0.332 | 39.62 | nucleus | |
| ZoPP2C11 | 285 | | 30876.51 | 4.87 | -0.198 | 31.49 | nucleus | |
| ZoPP2C21 | 286 | | 31767.97 | 4.97 | -0.214 | 36.92 | cytoplasm | |
| ZoPP2C39 | 296 | | 32620.58 | 5.71 | -0.333 | 41.74 | nucleus | |
| ZoPP2C18 | 306 | | 32921.51 | 4.91 | -0.384 | 35.84 | nucleus | |
| ZoPP2C17 | 315 | | 34453.68 | 4.9 | -0.345 | 37.22 | nucleus | |
| ZoPP2C14 | 316 | | 34865.06 | 5.13 | -0.412 | 42.65 | nucleus | |
| ZoPP2C45 | 342 | | 37230.51 | 9.17 | -0.167 | 42.05 | chloroplast | |
| ZoPP2C51 | 347 | | 38432.25 | 5.39 | -0.469 | 48.78 | nucleus | |
| ZoPP2C28 | 349 | | 37843.32 | 4.7 | -0.116 | 53.27 | nucleus | |
| ZoPP2C36 | 350 | | 38878.74 | 9 | -0.513 | 45.99 | chloroplast | |
| ZoPP2C26 | 355 | | 38528.92 | 6.73 | -0.211 | 60.64 | chloroplast | |
| ZoPP2C13 | 358 | | 39463.95 | 5.44 | -0.242 | 49.58 | nucleus | |
| ZoPP2C42 | 361 | | 39321.4 | 7.15 | -0.375 | 43.34 | nucleus | |
| ZoPP2C47 | 366 | | 40216.33 | 8.1 | -0.504 | 34 | nucleus | |
| ZoPP2C91 | 367 | | 40657.54 | 5.77 | -0.433 | 46.01 | nucleus | |
| ZoPP2C1 | 368 | | 40626.17 | 5.26 | -0.174 | 38.51 | extracellular | |
| ZoPP2C73 | 368 | | 40633.47 | 7.73 | -0.209 | 42.63 | nucleus | |
| ZoPP2C96 | 368 | | 41062.3 | 6.1 | -0.495 | 56.47 | nucleus | |
| ZoPP2C57 | 370 | | 41086.95 | 8.46 | -0.264 | 46.42 | mitochondrion | |
| ZoPP2C59 | 371 | | 41582.5 | 8.34 | -0.252 | 38.14 | nucleus | |
| ZoPP2C77 | 371 | | 41003.91 | 8.7 | -0.234 | 50.27 | nucleus | |
| ZoPP2C9 | 372 | | 39813.91 | 5.63 | -0.246 | 47.83 | nucleus | |
| ZoPP2C54 | 372 | | 41437.37 | 7.27 | -0.188 | 45.73 | nucleus | |
| ZoPP2C2 | 373 | | 40824.24 | 5.56 | -0.261 | 41.12 | nucleus | |
| ZoPP2C3 | 373 | | 41442.19 | 5.41 | -0.157 | 35.57 | extracellular | |
| ZoPP2C75 | 373 | | 41171.75 | 8.5 | -0.289 | 45.84 | chloroplast | |
| ZoPP2C69 | 374 | | 41568.43 | 8.53 | -0.275 | 45.12 | nucleus | |
| ZoPP2C35 | 375 | | 40357.6 | 5.73 | -0.264 | 54.54 | chloroplast | |
| ZoPP2C4 | 377 | | 40842.06 | 5.17 | -0.291 | 40.87 | nucleus | |
| ZoPP2C60 | 378 | | 42380 | 6.77 | -0.313 | 36.38 | nucleus | |
| ZoPP2C8 | 379 | | 40650.88 | 5.25 | -0.273 | 45.92 | chloroplast | |
| ZoPP2C6 | 380 | | 41286.81 | 5.71 | -0.284 | 59.7 | nucleus | |
| ZoPP2C72 | 381 | | 42837.78 | 6.24 | -0.18 | 39.28 | mitochondrion | |
| ZoPP2C15 | 382 | | 41059.37 | 5.39 | -0.256 | 45.74 | nucleus | |
| ZoPP2C71 | 382 | | 42445.48 | 8.2 | -0.208 | 37.03 | chloroplast | |
| ZoPP2C5 | 383 | | 41365.16 | 6.6 | -0.239 | 50.33 | nucleus | |
| ZoPP2C7 | 383 | | 40769.92 | 5.75 | -0.195 | 46.52 | nucleus | |
| ZoPP2C43 | 384 | | 41340.62 | 5.61 | -0.225 | 54.02 | chloroplast | |
| ZoPP2C41 | 390 | | 42941.17 | 6.96 | -0.01 | 43.43 | extracellular | |
| ZoPP2C67 | 390 | | 43125.15 | 7.27 | -0.152 | 43.85 | nucleus | |
| ZoPP2C58 | 391 | | 43641.81 | 8.95 | -0.259 | 46.87 | chloroplast | |
| ZoPP2C34 | 392 | | 42022.61 | 6.63 | -0.22 | 59.56 | chloroplast | |
| ZoPP2C62 | 392 | | 43830.08 | 8.19 | -0.233 | 42.47 | nucleus | |
| ZoPP2C20 | 396 | | 42997.46 | 4.87 | -0.132 | 50.61 | nucleus | |
| ZoPP2C63 | 398 | | 44277.39 | 7.69 | -0.283 | 45.93 | nucleus | |
| ZoPP2C64 | 398 | | 43357.77 | 4.76 | -0.067 | 36.15 | nucleus | |
| ZoPP2C29 | 402 | | 43498.98 | 4.7 | -0.158 | 44.39 | nucleus | |
| ZoPP2C80 | 406 | | 44899.22 | 8.48 | -0.249 | 43.24 | nucleus | |
| ZoPP2C52 | 407 | | 44844.76 | 8.47 | -0.425 | 46.5 | nucleus | |
| ZoPP2C10 | 408 | | 44325.09 | 4.9 | -0.085 | 53.77 | nucleus | |
| ZoPP2C70 | 410 | | 45247.35 | 6.9 | -0.242 | 48.45 | nucleus | |
| ZoPP2C32 | 411 | | 44563.71 | 6.07 | -0.214 | 34.63 | nucleus | |
| ZoPP2C56 | 411 | | 44120.47 | 5.69 | -0.173 | 42.42 | nucleus | |
| ZoPP2C78 | 412 | | 45527.65 | 6.68 | -0.209 | 53.54 | nucleus | |
| ZoPP2C50 | 414 | | 45699.53 | 7.59 | -0.456 | 39.1 | nucleus | |
| ZoPP2C12 | 415 | | 45349.42 | 5.97 | -0.172 | 62.9 | nucleus | |
| ZoPP2C90 | 417 | | 45833.98 | 5.09 | -0.236 | 43.11 | nucleus | |
| ZoPP2C55 | 418 | | 45905.54 | 6.05 | -0.411 | 36.04 | nucleus | |
| ZoPP2C86 | 418 | | 45075.46 | 5.79 | -0.171 | 41.21 | nucleus | |
| ZoPP2C22 | 420 | | 45264.03 | 5.45 | -0.396 | 59.2 | nucleus | |
| ZoPP2C88 | 423 | | 46067.5 | 5.66 | -0.206 | 43.83 | nucleus | |
| ZoPP2C92 | 425 | | 46026.95 | 4.9 | -0.239 | 40.12 | nucleus | |
| ZoPP2C23 | 430 | | 46573.32 | 8.7 | -0.191 | 56.35 | chloroplast | |
| ZoPP2C25 | 430 | | 48485.39 | 4.78 | -0.451 | 42.31 | nucleus | |
| ZoPP2C44 | 432 | | 47376.62 | 7.65 | -0.338 | 51.45 | chloroplast | |
| ZoPP2C27 | 445 | | 46905.74 | 5.14 | -0.188 | 64.85 | nucleus | |
| ZoPP2C33 | 445 | | 49274.87 | 6.58 | -0.431 | 52.38 | chloroplast | |
| ZoPP2C81 | 449 | | 49325.94 | 6.02 | -0.191 | 42.59 | nucleus | |
| ZoPP2C74 | 454 | | 49360.11 | 4.81 | -0.238 | 38.14 | nucleus | |
| ZoPP2C48 | 463 | | 50657.78 | 7.55 | -0.431 | 47.02 | chloroplast | |
| ZoPP2C68 | 472 | | 51694.14 | 5.48 | -0.365 | 44.9 | chloroplast | |
| ZoPP2C65 | 476 | | 52425.08 | 5.82 | -0.4 | 48.72 | chloroplast | |
| ZoPP2C66 | 476 | | 52509.24 | 5.82 | -0.392 | 48.7 | chloroplast | |
| ZoPP2C61 | 482 | | 52722.13 | 5.21 | -0.391 | 47.87 | nucleus | |
| ZoPP2C46 | 486 | | 53994 | 6.13 | -0.538 | 45 | nucleus | |
| ZoPP2C85 | 494 | | 53769.73 | 7.85 | -0.09 | 37.86 | extracellular | |
| ZoPP2C49 | 508 | | 55804.75 | 5.37 | -0.487 | 47.56 | nucleus | |
| ZoPP2C53 | 520 | | 57035.11 | 5.3 | -0.446 | 45.4 | nucleus | |
| ZoPP2C19 | 524 | | 56668.47 | 4.67 | -0.296 | 51.9 | nucleus | |
| ZoPP2C24 | 531 | | 57073.71 | 4.71 | -0.303 | 50.4 | nucleus | |
| ZoPP2C89 | 539 | | 58606.06 | 6.44 | -0.079 | 38.09 | endomembrane | |
| ZoPP2C76 | 589 | | 65093.35 | 5.54 | -0.293 | 60.9 | chloroplast | |
| ZoPP2C97 | 606 | | 64848.33 | 5.64 | -0.324 | 44.26 | mitochondrion | |
| ZoPP2C95 | 607 | | 65640.04 | 6.37 | -0.267 | 46.67 | nucleus | |
| ZoPP2C16 | 609 | | 67627.11 | 5.87 | -0.174 | 45.2 | chloroplast | |
| ZoPP2C83 | 640 | | 69695.78 | 5.74 | -0.423 | 44.3 | nucleus | |
| ZoPP2C31 | 653 | | 73374.83 | 5.87 | -0.541 | 42.15 | nucleus | |
| ZoPP2C82 | 661 | | 72159.29 | 5.98 | -0.309 | 46.62 | nucleus | |
| ZoPP2C93 | 663 | | 72564.26 | 6.33 | -0.346 | 50.69 | nucleus | |
| ZoPP2C87 | 667 | | 73463.93 | 5.55 | -0.402 | 47.71 | nucleus | |
| ZoPP2C84 | 728 | | 80571.29 | 6.49 | -0.562 | 47.6 | chloroplast | |
| ZoPP2C30 | 750 | | 81076.78 | 5.59 | -0.223 | 51 | nucleus | |
| ZoPP2C94 | 957 | | 106071.67 | 5.77 | -0.506 | 46.4 | nucleus | |
| ZoPP2C79 | 1068 | | 118732.42 | 5.26 | -0.224 | 42.04 | nucleus | |

aLength: amino acid length; MW: molecular weight; cpl: isoelectric point; dGRAVY: grand average of hydropathicity; fLoc: subcellular localization

Table S3 Segmental replication covariate gene pair Ka/Ks analysis of ZoPP2Cs

| Gene pair name | | Ka | Ks | Ka/Ks | Million years ago (MYA) | | Duplications type |
| --- | --- | --- | --- | --- | --- | --- | --- |
| ZoPP2C87 | ZoPP2C93 | 0.0623 | 0.3252 | 0.1917 | 25.021452 | segmental duplication | |
| ZoPP2C50 | ZoPP2C83 | 0.8369 | NaN | NaN | NaN | segmental duplication | |
| ZoPP2C76 | ZoPP2C59 | 0.6729 | NaN | NaN | NaN | segmental duplication | |
| ZoPP2C69 | ZoPP2C80 | 0.0643 | 0.6214 | 0.1035 | 47.80461462 | segmental duplication | |
| ZoPP2C14 | ZoPP2C47 | 0.7051 | NaN | NaN | NaN | segmental duplication | |
| ZoPP2C96 | ZoPP2C2 | 0.7930 | NaN | NaN | NaN | segmental duplication | |
| ZoPP2C91 | ZoPP2C72 | 0.8053 | NaN | NaN | NaN | segmental duplication | |
| ZoPP2C70 | ZoPP2C78 | 0.2156 | 0.4950 | 0.4357 | 38.07874454 | segmental duplication | |
| ZoPP2C41 | ZoPP2C74 | 0.8504 | NaN | NaN | NaN | segmental duplication | |
| ZoPP2C29 | ZoPP2C11 | 0.6250 | NaN | NaN | NaN | segmental duplication | |
| ZoPP2C81 | ZoPP2C67 | 0.0435 | 0.3925 | 0.1110 | 30.19273185 | segmental duplication | |
| ZoPP2C90 | ZoPP2C74 | 0.8368 | 1.8012 | 0.4646 | 138.5543328 | segmental duplication | |
| ZoPP2C34 | ZoPP2C35 | 0.093 | 0.3929 | 0.2382 | 30.22617769 | segmental duplication | |
| ZoPP2C82 | ZoPP2C22 | 1.0326 | NaN | NaN | NaN | segmental duplication | |
| ZoPP2C82 | ZoPP2C95 | 0.0837 | 0.3771 | 0.2220 | 29.01177977 | segmental duplication | |
| ZoPP2C95 | ZoPP2C52 | 0.7566 | NaN | NaN | NaN | segmental duplication | |
| ZoPP2C52 | ZoPP2C55 | 0.1455 | 0.6144 | 0.2368 | 47.264681 | segmental duplication | |
| ZoPP2C55 | ZoPP2C97 | 0.9769 | NaN | NaN | NaN | segmental duplication | |
| ZoPP2C42 | ZoPP2C49 | 0.7761 | NaN | NaN | NaN | segmental duplication | |
| ZoPP2C25 | ZoPP2C20 | 0.6941 | NaN | NaN | NaN | segmental duplication | |
| ZoPP2C33 | ZoPP2C10 | 0.7379 | NaN | NaN | NaN | segmental duplication | |
| ZoPP2C56 | ZoPP2C40 | 0.7336 | NaN | NaN | NaN | segmental duplication | |
| ZoPP2C8 | ZoPP2C7 | 0.0855 | 0.4103 | 0.2083 | 31.56761223 | segmental duplication | |
| ZoPP2C8 | ZoPP2C15 | 0.0058 | 0.0213 | 0.2755 | 1.644059462 | segmental duplication | |
| ZoPP2C66 | ZoPP2C65 | 0.0065 | 0.0231 | 0.2820 | 1.780424615 | segmental duplication | |
| ZoPP2C66 | ZoPP2C7 | 0.8614 | NaN | NaN | NaN | segmental duplication | |
| ZoPP2C8 | ZoPP2C65 | 0.8025 | 2.8843 | 0.2782 | 221.8693748 | segmental duplication | |
| ZoPP2C40 | ZoPP2C47 | 0.6982 | 2.3008 | 0.3034 | 176.9892205 | segmental duplication | |
| ZoPP2C37 | ZoPP2C47 | 0.7011 | 2.1122 | 0.3319 | 162.4845216 | segmental duplication | |
| ZoPP2C24 | ZoPP2C61 | 0.8463 | NaN | NaN | NaN | segmental duplication | |
| ZoPP2C24 | ZoPP2C19 | 0.1137 | 0.3705 | 0.3069 | 28.50453323 | segmental duplication | |
| ZoPP2C65 | ZoPP2C68 | 0.0903 | 0.4470 | 0.2022 | 34.38878508 | segmental duplication | |
| ZoPP2C15 | ZoPP2C7 | 0.0881 | 0.3983 | 0.2213 | 30.64472092 | segmental duplication | |

Ka, non-synonymous substitution rate; Ks, synonymous substitution rate; Ka/Ks, selection pressure ratio; Na, no result

Table S4 Duplicate gene pairs between different species

| Species | Gene pair name | |
| --- | --- | --- |
| Alfalfa vs Ginger | CM010649.1 | ZoPP2C64 |
| Cucumber vs Ginger | NC_026661.2 | ZoPP2C65 |
| Soybean vs Ginger | NC_038248.2 | ZoPP2C65 |
|  | NC_038251.2 | ZoPP2C64 |
| Banana vs Ginger | NC_025202.1 | ZoPP2C38 |
|  | NC_025203.1 | ZoPP2C75 |
|  | NC_025203.1 | ZoPP2C71 |
|  | NC_025203.1 | ZoPP2C73 |
|  | NC_025204.1 | ZoPP2C53 |
|  | NC_025204.1 | ZoPP2C10 |
|  | NC_025204.1 | ZoPP2C28 |
|  | NC_025204.1 | ZoPP2C71 |
|  | NC_025204.1 | ZoPP2C73 |
|  | NC_025204.1 | ZoPP2C64 |
|  | NC_025204.1 | ZoPP2C19 |
|  | NC_025205.1 | ZoPP2C16 |
|  | NC_025205.1 | ZoPP2C56 |
|  | NC_025205.1 | ZoPP2C45 |
|  | NC_025205.1 | ZoPP2C5 |
|  | NC_025205.1 | ZoPP2C6 |
|  | NC_025205.1 | ZoPP2C57 |
|  | NC_025205.1 | ZoPP2C14 |
|  | NC_025206.1 | ZoPP2C93 |
|  | NC_025206.1 | ZoPP2C95 |
|  | NC_025206.1 | ZoPP2C22 |
|  | NC_025206.1 | ZoPP2C43 |
|  | NC_025206.1 | ZoPP2C78 |
|  | NC_025206.1 | ZoPP2C88 |
|  | NC_025206.1 | ZoPP2C30 |
|  | NC_025206.1 | ZoPP2C82 |
|  | NC_025207.1 | ZoPP2C23 |
|  | NC_025207.1 | ZoPP2C55 |
|  | NC_025207.1 | ZoPP2C42 |
|  | NC_025207.1 | ZoPP2C46 |
|  | NC_025207.1 | ZoPP2C50 |
|  | NC_025207.1 | ZoPP2C1 |
|  | NC_025207.1 | ZoPP2C52 |
|  | NC_025208.1 | ZoPP2C67 |
|  | NC_025208.1 | ZoPP2C92 |
|  | NC_025208.1 | ZoPP2C2 |
|  | NC_025208.1 | ZoPP2C34 |
|  | NC_025208.1 | ZoPP2C14 |
|  | NC_025208.1 | ZoPP2C81 |
|  | NC_025208.1 | ZoPP2C4 |
|  | NC_025208.1 | ZoPP2C96 |
|  | NC_025208.1 | ZoPP2C35 |
|  | NC_025209.1 | ZoPP2C32 |
|  | NC_025209.1 | ZoPP2C44 |
|  | NC_025209.1 | ZoPP2C90 |
|  | NC_025209.1 | ZoPP2C64 |
|  | NC_025209.1 | ZoPP2C24 |
|  | NC_025209.1 | ZoPP2C52 |
|  | NC_025210.1 | ZoPP2C68 |
|  | NC_025210.1 | ZoPP2C65 |
|  | NC_025210.1 | ZoPP2C66 |
|  | NC_025210.1 | ZoPP2C55 |
|  | NC_025210.1 | ZoPP2C83 |
|  | NC_025210.1 | ZoPP2C50 |
|  | NC_025210.1 | ZoPP2C52 |
|  | NC_025210.1 | ZoPP2C47 |
|  | NC_025210.1 | ZoPP2C97 |
|  | NC_025211.1 | ZoPP2C2 |
|  | NC_025211.1 | ZoPP2C92 |
|  | NC_025211.1 | ZoPP2C44 |
|  | NC_025211.1 | ZoPP2C1 |
|  | NC_025211.1 | ZoPP2C76 |
|  | NC_025211.1 | ZoPP2C4 |
|  | NC_025211.1 | ZoPP2C26 |
|  | NC_025211.1 | ZoPP2C79 |
|  | NC_025211.1 | ZoPP2C79 |
|  | NC_025212.1 | ZoPP2C75 |
|  | NC_025212.1 | ZoPP2C30 |
|  | NC_025212.1 | ZoPP2C27 |

Table S5 ZoPP2C protein interaction network connection degree

| Gene NO. | Degree |
| --- | --- |
| ZoPP2C26 | 42 |
| ZoPP2C9 | 36 |
| ZoPP2C23 | 32 |
| ZoPP2C92 | 32 |
| ZoPP2C49 | 32 |
| ZoPP2C75 | 30 |
| ZoPP2C62 | 30 |
| ZoPP2C90 | 30 |
| ZoPP2C54 | 30 |
| ZoPP2C67 | 28 |
| ZoPP2C88 | 28 |
| ZoPP2C61 | 28 |
| ZoPP2C53 | 26 |
| ZoPP2C19 | 26 |
| ZoPP2C33 | 24 |
| ZoPP2C51 | 24 |
| ZoPP2C56 | 24 |
| ZoPP2C86 | 22 |
| ZoPP2C78 | 20 |
| ZoPP2C22 | 18 |
| ZoPP2C72 | 16 |
| ZoPP2C89 | 14 |
| ZoPP2C34 | 12 |
| ZoPP2C60 | 10 |
| ZoPP2C64 | 10 |
| ZoPP2C79 | 8 |
| ZoPP2C97 | 8 |
| ZoPP2C38 | 8 |
| ZoPP2C24 | 8 |
| ZoPP2C32 | 6 |
| ZoPP2C3 | 6 |
| ZoPP2C43 | 6 |
| ZoPP2C27 | 4 |
| ZoPP2C18 | 4 |
| ZoPP2C42 | 4 |
| ZoPP2C94 | 4 |
| ZoPP2C37 | 4 |
| ZoPP2C44 | 4 |
| ZoPP2C76 | 4 |
| ZoPP2C11 | 4 |
| ZoPP2C10 | 4 |
| ZoPP2C21 | 4 |
| ZoPP2C14 | 4 |
| ZoPP2C6 | 2 |
| ZoPP2C13 | 2 |
| ZoPP2C41 | 2 |

Table S6 ZoPP2Cs Protein Interaction Network KEGG Enrichment Analysis

| Pathway | Description | Strength | False discovery rate |
| --- | --- | --- | --- |
| map04075 | Plant hormone signal transduction | 0.89 | 0.00061 |
| map04016 | MAPK signaling pathway - plant | 1.04 | 0.0001 |
| map00940 | Phenylpropanoid biosynthesis | 0.99 | 0.00086 |

Table S7 ZoPP2C and potentially binding transcription factors (TFs)

| TF family | Gene NO. |
| --- | --- |
| AP2 | ZoPP2C41,ZoPP2C49,ZoPP2C27,ZoPP2C60,ZoPP2C40,ZoPP2C81,ZoPP2C54,ZoPP2C2,ZoPP2C32,ZoPP2C3,ZoPP2C53,ZoPP2C61,ZoPP2C17,ZoPP2C48,ZoPP2C14,ZoPP2C73,ZoPP2C20,ZoPP2C44,ZoPP2C37,ZoPP2C90,ZoPP2C12,ZoPP2C57,ZoPP2C59,ZoPP2C47,ZoPP2C46,ZoPP2C38,ZoPP2C1,ZoPP2C85,ZoPP2C70,ZoPP2C78,ZoPP2C9,ZoPP2C75,ZoPP2C35,ZoPP2C79,ZoPP2C43,ZoPP2C84,ZoPP2C31 ,ZoPP2C4,ZoPP2C25,ZoPP2C24,ZoPP2C42,ZoPP2C18,ZoPP2C16,ZoPP2C63,ZoPP2C67,ZoPP2C55,ZoPP2C86,ZoPP2C50,ZoPP2C95,ZoPP2C21,ZoPP2C10,ZoPP2C88,ZoPP2C82,ZoPP2C58,ZoPP2C29,ZoPP2C96,ZoPP2C71,ZoPP2C72,ZoPP2C74,ZoPP2C11,ZoPP2C34,ZoPP2C91,ZoPP2C66,ZoPP2C6,ZoPP2C19,ZoPP2C45,ZoPP2C76,ZoPP2C89,ZoPP2C94,ZoPP2C87,ZoPP2C36,ZoPP2C93,ZoPP2C28,ZoPP2C13,ZoPP2C26,ZoPP2C51,ZoPP2C64,ZoPP2C33,ZoPP2C92,ZoPP2C68,ZoPP2C15,ZoPP2C8,ZoPP2C39,ZoPP2C23,ZoPP2C77,ZoPP2C52,ZoPP2C65,ZoPP2C62,ZoPP2C30,ZoPP2C69,ZoPP2C7,ZoPP2C56 |
| ARF | ZoPP2C33,ZoPP2C4,ZoPP2C7,ZoPP2C49,ZoPP2C42,ZoPP2C1,ZoPP2C61,ZoPP2C36,ZoPP2C95,ZoPP2C82,ZoPP2C27,ZoPP2C46,ZoPP2C76,ZoPP2C97,ZoPP2C25,ZoPP2C47,ZoPP2C85,ZoPP2C32,ZoPP2C55,ZoPP2C62,ZoPP2C63,ZoPP2C80,ZoPP2C60,ZoPP2C50,ZoPP2C84,ZoPP2C83,ZoPP2C88,ZoPP2C86,ZoPP2C26,ZoPP2C21,ZoPP2C9,ZoPP2C90,ZoPP2C30,ZoPP2C96,ZoPP2C75,ZoPP2C89,ZoPP2C94,ZoPP2C12,ZoPP2C81,ZoPP2C92,ZoPP2C56,ZoPP2C17,ZoPP2C87,ZoPP2C58,ZoPP2C44,ZoPP2C14,ZoPP2C41,ZoPP2C3,ZoPP2C29,ZoPP2C73,ZoPP2C79,ZoPP2C67,ZoPP2C71,ZoPP2C78,ZoPP2C37,ZoPP2C19,ZoPP2C65,ZoPP2C66,ZoPP2C74,ZoPP2C43,ZoPP2C38,ZoPP2C45,ZoPP2C6,ZoPP2C52,ZoPP2C69,ZoPP2C18,ZoPP2C68,ZoPP2C5,ZoPP2C70,ZoPP2C54,ZoPP2C8,ZoPP2C15,ZoPP2C31,ZoPP2C20,ZoPP2C39,ZoPP2C40,ZoPP2C2,ZoPP2C64,ZoPP2C72,ZoPP2C77,ZoPP2C13,ZoPP2C59,ZoPP2C48,ZoPP2C10,ZoPP2C11,ZoPP2C51,ZoPP2C91,ZoPP2C23,ZoPP2C93,ZoPP2C16,ZoPP2C34,ZoPP2C53 |
| ARR-B | ZoPP2C55,ZoPP2C50,ZoPP2C49,ZoPP2C94,ZoPP2C16,ZoPP2C72,ZoPP2C27,ZoPP2C74,ZoPP2C45,ZoPP2C65,ZoPP2C66,ZoPP2C95,ZoPP2C18,ZoPP2C80,ZoPP2C85,ZoPP2C86,ZoPP2C67,ZoPP2C89,ZoPP2C83,ZoPP2C47,ZoPP2C61,ZoPP2C32,ZoPP2C69,ZoPP2C79,ZoPP2C73,ZoPP2C58,ZoPP2C36,ZoPP2C11,ZoPP2C62,ZoPP2C31,ZoPP2C68,ZoPP2C13,ZoPP2C70,ZoPP2C46,ZoPP2C2,ZoPP2C41,ZoPP2C84,ZoPP2C82,ZoPP2C17,ZoPP2C12,ZoPP2C3,ZoPP2C20,ZoPP2C6,ZoPP2C78,ZoPP2C75,ZoPP2C1,ZoPP2C25,ZoPP2C44,ZoPP2C52,ZoPP2C9,ZoPP2C63,ZoPP2C59,ZoPP2C76,ZoPP2C90,ZoPP2C43,ZoPP2C48,ZoPP2C33 |
| B3 | ZoPP2C81,ZoPP2C8,ZoPP2C76,ZoPP2C47,ZoPP2C71,ZoPP2C49,ZoPP2C36,ZoPP2C10,ZoPP2C79,ZoPP2C2,ZoPP2C69,ZoPP2C87,ZoPP2C85,ZoPP2C90,ZoPP2C72,ZoPP2C9,ZoPP2C1,ZoPP2C25,ZoPP2C21,ZoPP2C11,ZoPP2C27,ZoPP2C58,ZoPP2C84,ZoPP2C48,ZoPP2C82,ZoPP2C28,ZoPP2C30,ZoPP2C45,ZoPP2C75,ZoPP2C4,ZoPP2C46,ZoPP2C54,ZoPP2C17,ZoPP2C89,ZoPP2C97,ZoPP2C29,ZoPP2C31,ZoPP2C32,ZoPP2C18,ZoPP2C68,ZoPP2C3,ZoPP2C16,ZoPP2C50,ZoPP2C43,ZoPP2C93,ZoPP2C42,ZoPP2C67,ZoPP2C94,ZoPP2C62,ZoPP2C91,ZoPP2C26,ZoPP2C7,ZoPP2C14,ZoPP2C38,ZoPP2C60,ZoPP2C55,ZoPP2C52,ZoPP2C40,ZoPP2C37,ZoPP2C44,ZoPP2C88,ZoPP2C61,ZoPP2C24,ZoPP2C13,ZoPP2C19,ZoPP2C59,ZoPP2C63,ZoPP2C74,ZoPP2C92,ZoPP2C73,ZoPP2C41,ZoPP2C95,ZoPP2C78,ZoPP2C65,ZoPP2C66,ZoPP2C20,ZoPP2C96,ZoPP2C12,ZoPP2C53,ZoPP2C86,ZoPP2C6,ZoPP2C80,ZoPP2C5,ZoPP2C33,ZoPP2C83,ZoPP2C70 |
| BBR-BPC | ZoPP2C25,ZoPP2C85,ZoPP2C47,ZoPP2C4,ZoPP2C31,ZoPP2C51,ZoPP2C91,ZoPP2C49,ZoPP2C44,ZoPP2C86,ZoPP2C60,ZoPP2C75,ZoPP2C59,ZoPP2C90,ZoPP2C14,ZoPP2C27,ZoPP2C17,ZoPP2C41,ZoPP2C32,ZoPP2C76,ZoPP2C88,ZoPP2C61,ZoPP2C20,ZoPP2C46,ZoPP2C50,ZoPP2C3,ZoPP2C38,ZoPP2C84,ZoPP2C19,ZoPP2C89,ZoPP2C48,ZoPP2C55,ZoPP2C79,ZoPP2C36,ZoPP2C92,ZoPP2C10,ZoPP2C72,ZoPP2C67,ZoPP2C87,ZoPP2C57,ZoPP2C16,ZoPP2C69,ZoPP2C1,ZoPP2C12,ZoPP2C39,ZoPP2C77,ZoPP2C2,ZoPP2C34,ZoPP2C21,ZoPP2C9,ZoPP2C81,ZoPP2C45,ZoPP2C70,ZoPP2C56,ZoPP2C63,ZoPP2C18,ZoPP2C11,ZoPP2C94,ZoPP2C13,ZoPP2C78,ZoPP2C24,ZoPP2C6,ZoPP2C74,ZoPP2C33,ZoPP2C62,ZoPP2C66,ZoPP2C65,ZoPP2C71,ZoPP2C54,ZoPP2C42,ZoPP2C43,ZoPP2C26,ZoPP2C30,ZoPP2C23,ZoPP2C22,ZoPP2C40,ZoPP2C82,ZoPP2C93,ZoPP2C53,ZoPP2C68,ZoPP2C7,ZoPP2C96,ZoPP2C15,ZoPP2C8,ZoPP2C58,ZoPP2C28,ZoPP2C37,ZoPP2C83,ZoPP2C29,ZoPP2C35 |
| BES1 | ZoPP2C14,ZoPP2C97,ZoPP2C47,ZoPP2C17,ZoPP2C23,ZoPP2C27,ZoPP2C26,ZoPP2C54,ZoPP2C81,ZoPP2C30,ZoPP2C32,ZoPP2C72,ZoPP2C86,ZoPP2C89,ZoPP2C7,ZoPP2C1,ZoPP2C95,ZoPP2C82,ZoPP2C83,ZoPP2C67,ZoPP2C61,ZoPP2C19,ZoPP2C36,ZoPP2C24,ZoPP2C31,ZoPP2C69,ZoPP2C91,ZoPP2C20,ZoPP2C38,ZoPP2C85,ZoPP2C90,ZoPP2C59,ZoPP2C25,ZoPP2C45,ZoPP2C12,ZoPP2C18,ZoPP2C41,ZoPP2C51,ZoPP2C2,ZoPP2C52,ZoPP2C63,ZoPP2C56 |
| bHLH | ZoPP2C90,ZoPP2C23,ZoPP2C47,ZoPP2C70,ZoPP2C97,ZoPP2C25,ZoPP2C29,ZoPP2C18,ZoPP2C54,ZoPP2C49,ZoPP2C83,ZoPP2C31,ZoPP2C32,ZoPP2C14,ZoPP2C20,ZoPP2C27,ZoPP2C2,ZoPP2C1,ZoPP2C85,ZoPP2C44,ZoPP2C88,ZoPP2C17,ZoPP2C41,ZoPP2C26,ZoPP2C30,ZoPP2C7,ZoPP2C52,ZoPP2C93,ZoPP2C87,ZoPP2C84,ZoPP2C38,ZoPP2C78,ZoPP2C21,ZoPP2C81,ZoPP2C50,ZoPP2C40,ZoPP2C37,ZoPP2C76,ZoPP2C11,ZoPP2C46,ZoPP2C75,ZoPP2C42,ZoPP2C86,ZoPP2C80,ZoPP2C94,ZoPP2C19,ZoPP2C3,ZoPP2C61,ZoPP2C71,ZoPP2C55,ZoPP2C59,ZoPP2C45,ZoPP2C62,ZoPP2C15,ZoPP2C68,ZoPP2C65,ZoPP2C66,ZoPP2C67,ZoPP2C79,ZoPP2C36,ZoPP2C82,ZoPP2C63,ZoPP2C51,ZoPP2C89,ZoPP2C95,ZoPP2C74,ZoPP2C58,ZoPP2C53,ZoPP2C4,ZoPP2C56,ZoPP2C16,ZoPP2C22,ZoPP2C92,ZoPP2C60,ZoPP2C91,ZoPP2C48,ZoPP2C96,ZoPP2C77,ZoPP2C5,ZoPP2C12,ZoPP2C6 |
| bZIP | ZoPP2C63,ZoPP2C47,ZoPP2C57,ZoPP2C49,ZoPP2C44,ZoPP2C1,ZoPP2C90,ZoPP2C23,ZoPP2C66,ZoPP2C31,ZoPP2C85,ZoPP2C39,ZoPP2C28,ZoPP2C27,ZoPP2C30,ZoPP2C93,ZoPP2C76,ZoPP2C33,ZoPP2C58,ZoPP2C2,ZoPP2C60,ZoPP2C59,ZoPP2C87,ZoPP2C73,ZoPP2C20,ZoPP2C68,ZoPP2C65,ZoPP2C10,ZoPP2C96,ZoPP2C71,ZoPP2C52,ZoPP2C53,ZoPP2C61,ZoPP2C86,ZoPP2C77,ZoPP2C3,ZoPP2C79,ZoPP2C17,ZoPP2C36,ZoPP2C91,ZoPP2C70,ZoPP2C9,ZoPP2C25,ZoPP2C16,ZoPP2C32,ZoPP2C78,ZoPP2C84,ZoPP2C5,ZoPP2C81,ZoPP2C45,ZoPP2C69,ZoPP2C48,ZoPP2C80,ZoPP2C19,ZoPP2C92,ZoPP2C56,ZoPP2C72,ZoPP2C12,ZoPP2C88,ZoPP2C50,ZoPP2C75,ZoPP2C29,ZoPP2C4,ZoPP2C46,ZoPP2C67,ZoPP2C95,ZoPP2C82,ZoPP2C14,ZoPP2C8,ZoPP2C15,ZoPP2C42,ZoPP2C51,ZoPP2C54,ZoPP2C22,ZoPP2C89,ZoPP2C41,ZoPP2C11,ZoPP2C94,ZoPP2C35,ZoPP2C24,ZoPP2C38,ZoPP2C43,ZoPP2C83,ZoPP2C26,ZoPP2C97,ZoPP2C13,ZoPP2C62,ZoPP2C40,ZoPP2C37,ZoPP2C21,ZoPP2C6,ZoPP2C18,ZoPP2C7 |
| C2H2 | ZoPP2C83,ZoPP2C97,ZoPP2C96,ZoPP2C4,ZoPP2C90,ZoPP2C26,ZoPP2C56,ZoPP2C16,ZoPP2C28,ZoPP2C9,ZoPP2C32,ZoPP2C60,ZoPP2C84,ZoPP2C51,ZoPP2C3,ZoPP2C95,ZoPP2C82,ZoPP2C57,ZoPP2C89,ZoPP2C85,ZoPP2C36,ZoPP2C52,ZoPP2C69,ZoPP2C61,ZoPP2C47,ZoPP2C71,ZoPP2C35,ZoPP2C25,ZoPP2C92,ZoPP2C73,ZoPP2C54,ZoPP2C43,ZoPP2C48,ZoPP2C80,ZoPP2C2,ZoPP2C24,ZoPP2C78,ZoPP2C15,ZoPP2C8,ZoPP2C22,ZoPP2C45,ZoPP2C27,ZoPP2C65,ZoPP2C66,ZoPP2C72,ZoPP2C94,ZoPP2C29,ZoPP2C88,ZoPP2C53,ZoPP2C23,ZoPP2C86,ZoPP2C49,ZoPP2C74,ZoPP2C87,ZoPP2C33,ZoPP2C14,ZoPP2C18,ZoPP2C81,ZoPP2C93,ZoPP2C64,ZoPP2C76,ZoPP2C31,ZoPP2C38,ZoPP2C46,ZoPP2C13,ZoPP2C10,ZoPP2C79,ZoPP2C62,ZoPP2C34,ZoPP2C11,ZoPP2C67,ZoPP2C50,ZoPP2C21,ZoPP2C55,ZoPP2C41,ZoPP2C44,ZoPP2C63,ZoPP2C59,ZoPP2C1,ZoPP2C70,ZoPP2C17,ZoPP2C68,ZoPP2C20,ZoPP2C40,ZoPP2C37,ZoPP2C30,ZoPP2C12,ZoPP2C19,ZoPP2C42,ZoPP2C91,ZoPP2C5,ZoPP2C75,ZoPP2C6,ZoPP2C7,ZoPP2C77,ZoPP2C39,ZoPP2C58 |
| C3H | ZoPP2C78,ZoPP2C47,ZoPP2C81,ZoPP2C85,ZoPP2C27,ZoPP2C79,ZoPP2C15,ZoPP2C8,ZoPP2C17,ZoPP2C36,ZoPP2C31,ZoPP2C18,ZoPP2C49,ZoPP2C67,ZoPP2C93,ZoPP2C3,ZoPP2C96,ZoPP2C70,ZoPP2C53,ZoPP2C92,ZoPP2C61,ZoPP2C13,ZoPP2C1,ZoPP2C59,ZoPP2C50,ZoPP2C19,ZoPP2C39,ZoPP2C48,ZoPP2C4,ZoPP2C87,ZoPP2C60,ZoPP2C25,ZoPP2C10,ZoPP2C32,ZoPP2C42,ZoPP2C86,ZoPP2C55,ZoPP2C38,ZoPP2C44,ZoPP2C72,ZoPP2C40,ZoPP2C37,ZoPP2C29,ZoPP2C57,ZoPP2C90,ZoPP2C16,ZoPP2C75,ZoPP2C88,ZoPP2C12,ZoPP2C71,ZoPP2C33,ZoPP2C54,ZoPP2C2,ZoPP2C41,ZoPP2C30,ZoPP2C63,ZoPP2C74,ZoPP2C69,ZoPP2C65,ZoPP2C66,ZoPP2C20,ZoPP2C26,ZoPP2C7,ZoPP2C76,ZoPP2C14,ZoPP2C89,ZoPP2C58,ZoPP2C11,ZoPP2C6,ZoPP2C28,ZoPP2C46,ZoPP2C95,ZoPP2C23,ZoPP2C91,ZoPP2C68,ZoPP2C94,ZoPP2C21 |
| CAMTA | ZoPP2C69,ZoPP2C36,ZoPP2C35,ZoPP2C34,ZoPP2C49,ZoPP2C65,ZoPP2C89,ZoPP2C47,ZoPP2C52,ZoPP2C22,ZoPP2C26,ZoPP2C72,ZoPP2C30,ZoPP2C7,ZoPP2C14,ZoPP2C80,ZoPP2C44,ZoPP2C90,ZoPP2C76,ZoPP2C97,ZoPP2C54,ZoPP2C79,ZoPP2C23,ZoPP2C32,ZoPP2C10,ZoPP2C45,ZoPP2C86 |
| CPP | ZoPP2C55,ZoPP2C27,ZoPP2C17,ZoPP2C32,ZoPP2C47,ZoPP2C75,ZoPP2C3,ZoPP2C59,ZoPP2C14,ZoPP2C21,ZoPP2C63,ZoPP2C85,ZoPP2C67,ZoPP2C10,ZoPP2C25,ZoPP2C61,ZoPP2C11,ZoPP2C86,ZoPP2C19,ZoPP2C36,ZoPP2C49,ZoPP2C92,ZoPP2C57,ZoPP2C79,ZoPP2C29,ZoPP2C40,ZoPP2C37,ZoPP2C74,ZoPP2C88,ZoPP2C71,ZoPP2C50,ZoPP2C38,ZoPP2C91,ZoPP2C41,ZoPP2C52,ZoPP2C90,ZoPP2C62,ZoPP2C81,ZoPP2C4,ZoPP2C2,ZoPP2C76,ZoPP2C96,ZoPP2C18,ZoPP2C97,ZoPP2C1,ZoPP2C70,ZoPP2C48,ZoPP2C78,ZoPP2C54,ZoPP2C94,ZoPP2C20,ZoPP2C43,ZoPP2C30,ZoPP2C16,ZoPP2C31,ZoPP2C26,ZoPP2C45,ZoPP2C87,ZoPP2C9,ZoPP2C46,ZoPP2C7,ZoPP2C8,ZoPP2C15,ZoPP2C44,ZoPP2C12,ZoPP2C39,ZoPP2C60,ZoPP2C72,ZoPP2C24,ZoPP2C77,ZoPP2C34,ZoPP2C93,ZoPP2C42,ZoPP2C89,ZoPP2C84,ZoPP2C53,ZoPP2C22,ZoPP2C6 |
| Dof | ZoPP2C60,ZoPP2C3,ZoPP2C27,ZoPP2C49,ZoPP2C32,ZoPP2C54,ZoPP2C2,ZoPP2C41,ZoPP2C70,ZoPP2C48,ZoPP2C85,ZoPP2C53,ZoPP2C14,ZoPP2C57,ZoPP2C61,ZoPP2C13,ZoPP2C90,ZoPP2C9,ZoPP2C78,ZoPP2C47,ZoPP2C1,ZoPP2C96,ZoPP2C73,ZoPP2C79,ZoPP2C18,ZoPP2C38,ZoPP2C92,ZoPP2C81,ZoPP2C67,ZoPP2C42,ZoPP2C36,ZoPP2C44,ZoPP2C17,ZoPP2C40,ZoPP2C37,ZoPP2C16,ZoPP2C12,ZoPP2C88,ZoPP2C59,ZoPP2C76,ZoPP2C24,ZoPP2C93,ZoPP2C55,ZoPP2C26,ZoPP2C7,ZoPP2C25,ZoPP2C31,ZoPP2C19,ZoPP2C46,ZoPP2C86,ZoPP2C63,ZoPP2C4,ZoPP2C82,ZoPP2C87,ZoPP2C72,ZoPP2C43,ZoPP2C6,ZoPP2C75,ZoPP2C89,ZoPP2C20,ZoPP2C50,ZoPP2C68,ZoPP2C23,ZoPP2C29,ZoPP2C15,ZoPP2C8,ZoPP2C58,ZoPP2C74,ZoPP2C94,ZoPP2C35,ZoPP2C33,ZoPP2C34,ZoPP2C71,ZoPP2C28,ZoPP2C11,ZoPP2C39,ZoPP2C22,ZoPP2C45,ZoPP2C69,ZoPP2C10,ZoPP2C21,ZoPP2C62,ZoPP2C51,ZoPP2C84,ZoPP2C64,ZoPP2C5,ZoPP2C30,ZoPP2C97,ZoPP2C65,ZoPP2C66,ZoPP2C95,ZoPP2C91,ZoPP2C52,ZoPP2C80,ZoPP2C77 |
| E2F/DP | ZoPP2C47,ZoPP2C49,ZoPP2C56,ZoPP2C92,ZoPP2C12,ZoPP2C90,ZoPP2C53,ZoPP2C1,ZoPP2C87,ZoPP2C25,ZoPP2C18,ZoPP2C46,ZoPP2C3,ZoPP2C86,ZoPP2C85,ZoPP2C79,ZoPP2C26,ZoPP2C89,ZoPP2C19,ZoPP2C67,ZoPP2C74,ZoPP2C69,ZoPP2C95,ZoPP2C42,ZoPP2C32,ZoPP2C60,ZoPP2C55,ZoPP2C17,ZoPP2C31,ZoPP2C24,ZoPP2C70,ZoPP2C48,ZoPP2C36,ZoPP2C73,ZoPP2C21,ZoPP2C27 |
| EIL | ZoPP2C74ZoPP2C47ZoPP2C27ZoPP2C13ZoPP2C49ZoPP2C38ZoPP2C45ZoPP2C94ZoPP2C51ZoPP2C20ZoPP2C79ZoPP2C90ZoPP2C32ZoPP2C58ZoPP2C2ZoPP2C65ZoPP2C66ZoPP2C1ZoPP2C89ZoPP2C59ZoPP2C25ZoPP2C16ZoPP2C81ZoPP2C61ZoPP2C36ZoPP2C24ZoPP2C76ZoPP2C21ZoPP2C29ZoPP2C62ZoPP2C88ZoPP2C60ZoPP2C19ZoPP2C84ZoPP2C4ZoPP2C70ZoPP2C53ZoPP2C42ZoPP2C31ZoPP2C67ZoPP2C63ZoPP2C93ZoPP2C52ZoPP2C69ZoPP2C71ZoPP2C85ZoPP2C50ZoPP2C86ZoPP2C87ZoPP2C75ZoPP2C78ZoPP2C54ZoPP2C37ZoPP2C40ZoPP2C7ZoPP2C3ZoPP2C30ZoPP2C11ZoPP2C26ZoPP2C17ZoPP2C39ZoPP2C48ZoPP2C33ZoPP2C9ZoPP2C14ZoPP2C46ZoPP2C6ZoPP2C18ZoPP2C72ZoPP2C34ZoPP2C44ZoPP2C15ZoPP2C8 |
| ERF | ZoPP2C82,ZoPP2C16,ZoPP2C97,ZoPP2C26,ZoPP2C65,ZoPP2C66,ZoPP2C28,ZoPP2C35,ZoPP2C61,ZoPP2C74,ZoPP2C49,ZoPP2C71,ZoPP2C23,ZoPP2C95,ZoPP2C64,ZoPP2C30,ZoPP2C67,ZoPP2C56,ZoPP2C7,ZoPP2C4,ZoPP2C93,ZoPP2C15,ZoPP2C8,ZoPP2C73,ZoPP2C27,ZoPP2C47,ZoPP2C34,ZoPP2C3,ZoPP2C33,ZoPP2C44,ZoPP2C10,ZoPP2C76,ZoPP2C48,ZoPP2C53,ZoPP2C55,ZoPP2C13,ZoPP2C87,ZoPP2C59,ZoPP2C9,ZoPP2C22,ZoPP2C69,ZoPP2C72,ZoPP2C68,ZoPP2C50,ZoPP2C77,ZoPP2C31,ZoPP2C85,ZoPP2C43,ZoPP2C57,ZoPP2C52,ZoPP2C21,ZoPP2C96,ZoPP2C83,ZoPP2C19,ZoPP2C80,ZoPP2C11,ZoPP2C2,ZoPP2C91,ZoPP2C84,ZoPP2C29,ZoPP2C60,ZoPP2C36,ZoPP2C79,ZoPP2C89,ZoPP2C20,ZoPP2C75,ZoPP2C54,ZoPP2C41,ZoPP2C40,ZoPP2C37,ZoPP2C24,ZoPP2C14,ZoPP2C88,ZoPP2C78,ZoPP2C45,ZoPP2C90,ZoPP2C46,ZoPP2C32,ZoPP2C51,ZoPP2C5,ZoPP2C42,ZoPP2C25,ZoPP2C70,ZoPP2C92,ZoPP2C12,ZoPP2C94,ZoPP2C17,ZoPP2C86,ZoPP2C39,ZoPP2C58,ZoPP2C38,ZoPP2C63,ZoPP2C18,ZoPP2C1,ZoPP2C6,ZoPP2C81,ZoPP2C62 |
| G2-like | ZoPP2C12,ZoPP2C54,ZoPP2C27,ZoPP2C85,ZoPP2C86,ZoPP2C16,ZoPP2C59,ZoPP2C47,ZoPP2C90,ZoPP2C36,ZoPP2C81,ZoPP2C4,ZoPP2C74,ZoPP2C1,ZoPP2C49,ZoPP2C68,ZoPP2C79,ZoPP2C2,ZoPP2C18,ZoPP2C67,ZoPP2C76,ZoPP2C78,ZoPP2C41,ZoPP2C75,ZoPP2C48,ZoPP2C60,ZoPP2C46,ZoPP2C51,ZoPP2C13,ZoPP2C88,ZoPP2C21,ZoPP2C3,ZoPP2C52,ZoPP2C70,ZoPP2C94,ZoPP2C63,ZoPP2C10,ZoPP2C40,ZoPP2C37,ZoPP2C11,ZoPP2C58,ZoPP2C25,ZoPP2C20,ZoPP2C24,ZoPP2C19,ZoPP2C39,ZoPP2C53,ZoPP2C45,ZoPP2C42,ZoPP2C26,ZoPP2C29,ZoPP2C15,ZoPP2C8,ZoPP2C32,ZoPP2C55,ZoPP2C92,ZoPP2C95,ZoPP2C34,ZoPP2C80,ZoPP2C71,ZoPP2C6,ZoPP2C82,ZoPP2C84,ZoPP2C30,ZoPP2C31,ZoPP2C9,ZoPP2C66,ZoPP2C7,ZoPP2C17,ZoPP2C89,ZoPP2C61,ZoPP2C62,ZoPP2C14,ZoPP2C93,ZoPP2C44,ZoPP2C5,ZoPP2C38,ZoPP2C28,ZoPP2C73,ZoPP2C57,ZoPP2C72,ZoPP2C97,ZoPP2C50,ZoPP2C65,ZoPP2C87,ZoPP2C69,ZoPP2C35,ZoPP2C96,ZoPP2C77,ZoPP2C56,ZoPP2C22,ZoPP2C83 |
| GATA | ZoPP2C36ZoPP2C80ZoPP2C70ZoPP2C83ZoPP2C97ZoPP2C90ZoPP2C94ZoPP2C76ZoPP2C61ZoPP2C92ZoPP2C11ZoPP2C72ZoPP2C3ZoPP2C29ZoPP2C69ZoPP2C82ZoPP2C26ZoPP2C24ZoPP2C27ZoPP2C19ZoPP2C91ZoPP2C79ZoPP2C25ZoPP2C53ZoPP2C81ZoPP2C67ZoPP2C16ZoPP2C4ZoPP2C12ZoPP2C1ZoPP2C18ZoPP2C30ZoPP2C17ZoPP2C6ZoPP2C60ZoPP2C85ZoPP2C32ZoPP2C95ZoPP2C71ZoPP2C86ZoPP2C96ZoPP2C63ZoPP2C75ZoPP2C46ZoPP2C49ZoPP2C35ZoPP2C55ZoPP2C38ZoPP2C51ZoPP2C41ZoPP2C45ZoPP2C20ZoPP2C66ZoPP2C65ZoPP2C2ZoPP2C44ZoPP2C78ZoPP2C54ZoPP2C84ZoPP2C56ZoPP2C50ZoPP2C89ZoPP2C10ZoPP2C33ZoPP2C74ZoPP2C9ZoPP2C87ZoPP2C34ZoPP2C22ZoPP2C68ZoPP2C39ZoPP2C62ZoPP2C23ZoPP2C88ZoPP2C43ZoPP2C93ZoPP2C58ZoPP2C21ZoPP2C31ZoPP2C15ZoPP2C8ZoPP2C7ZoPP2C77ZoPP2C28ZoPP2C57ZoPP2C13ZoPP2C48ZoPP2C64ZoPP2C37ZoPP2C40ZoPP2C47ZoPP2C14ZoPP2C59ZoPP2C73ZoPP2C42ZoPP2C5ZoPP2C52 |
| HD-ZIP | ZoPP2C90,ZoPP2C24,ZoPP2C19,ZoPP2C1,ZoPP2C63,ZoPP2C25,ZoPP2C76,ZoPP2C27,ZoPP2C89,ZoPP2C83,ZoPP2C67,ZoPP2C93,ZoPP2C85,ZoPP2C41,ZoPP2C38,ZoPP2C49,ZoPP2C45,ZoPP2C62,ZoPP2C36,ZoPP2C10,ZoPP2C31,ZoPP2C71,ZoPP2C54,ZoPP2C46,ZoPP2C74,ZoPP2C30,ZoPP2C72,ZoPP2C32,ZoPP2C75,ZoPP2C87,ZoPP2C52,ZoPP2C53,ZoPP2C3,ZoPP2C94,ZoPP2C61,ZoPP2C78,ZoPP2C84,ZoPP2C14,ZoPP2C73,ZoPP2C88,ZoPP2C47,ZoPP2C29,ZoPP2C81,ZoPP2C68,ZoPP2C59,ZoPP2C40,ZoPP2C37,ZoPP2C48,ZoPP2C16,ZoPP2C17,ZoPP2C58,ZoPP2C86,ZoPP2C9,ZoPP2C4,ZoPP2C55,ZoPP2C95,ZoPP2C82,ZoPP2C18,ZoPP2C50,ZoPP2C92,ZoPP2C69,ZoPP2C11,ZoPP2C20,ZoPP2C97,ZoPP2C2,ZoPP2C65,ZoPP2C66,ZoPP2C79,ZoPP2C80,ZoPP2C6,ZoPP2C96,ZoPP2C13,ZoPP2C60,ZoPP2C70,ZoPP2C5,ZoPP2C28,ZoPP2C39,ZoPP2C26,ZoPP2C21,ZoPP2C44,ZoPP2C15,ZoPP2C34,ZoPP2C8,ZoPP2C43,ZoPP2C22,ZoPP2C91,ZoPP2C51,ZoPP2C42,ZoPP2C77,ZoPP2C57,ZoPP2C12 |
| HSF | ZoPP2C75,ZoPP2C31,ZoPP2C67,ZoPP2C17,ZoPP2C60,ZoPP2C26,ZoPP2C89,ZoPP2C85,ZoPP2C54,ZoPP2C13,ZoPP2C57,ZoPP2C1,ZoPP2C63,ZoPP2C62,ZoPP2C58,ZoPP2C25,ZoPP2C19,ZoPP2C87,ZoPP2C76,ZoPP2C88,ZoPP2C30,ZoPP2C47,ZoPP2C53,ZoPP2C68,ZoPP2C27,ZoPP2C36,ZoPP2C79,ZoPP2C40,ZoPP2C37,ZoPP2C59,ZoPP2C38,ZoPP2C3,ZoPP2C49,ZoPP2C83,ZoPP2C32,ZoPP2C9,ZoPP2C70,ZoPP2C72,ZoPP2C78,ZoPP2C48,ZoPP2C24,ZoPP2C46,ZoPP2C29,ZoPP2C2,ZoPP2C39,ZoPP2C4,ZoPP2C12,ZoPP2C92,ZoPP2C28,ZoPP2C52,ZoPP2C61,ZoPP2C90,ZoPP2C81,ZoPP2C20,ZoPP2C43,ZoPP2C96,ZoPP2C55,ZoPP2C50,ZoPP2C73,ZoPP2C86,ZoPP2C11,ZoPP2C94,ZoPP2C14,ZoPP2C74,ZoPP2C56,ZoPP2C18,ZoPP2C42,ZoPP2C34,ZoPP2C97,ZoPP2C51,ZoPP2C6,ZoPP2C22,ZoPP2C44,ZoPP2C91,ZoPP2C21,ZoPP2C35,ZoPP2C5,ZoPP2C71,ZoPP2C93,ZoPP2C10,ZoPP2C77,ZoPP2C82,ZoPP2C64,ZoPP2C65,ZoPP2C66,ZoPP2C41,ZoPP2C8,ZoPP2C15,ZoPP2C69,ZoPP2C84,ZoPP2C95,ZoPP2C16 |
| LBD | ZoPP2C95ZoPP2C56ZoPP2C9ZoPP2C90ZoPP2C87ZoPP2C84ZoPP2C83ZoPP2C14ZoPP2C79ZoPP2C58ZoPP2C93ZoPP2C20ZoPP2C53ZoPP2C61ZoPP2C50ZoPP2C89ZoPP2C30ZoPP2C85ZoPP2C35ZoPP2C78ZoPP2C82ZoPP2C65ZoPP2C21ZoPP2C86ZoPP2C47ZoPP2C52ZoPP2C31ZoPP2C36ZoPP2C62ZoPP2C23ZoPP2C33ZoPP2C3ZoPP2C26ZoPP2C27ZoPP2C97ZoPP2C72ZoPP2C32ZoPP2C1ZoPP2C29ZoPP2C22ZoPP2C63ZoPP2C80ZoPP2C25ZoPP2C38ZoPP2C18ZoPP2C91ZoPP2C51 |
| LFY | ZoPP2C70,ZoPP2C82,ZoPP2C90,ZoPP2C47,ZoPP2C95,ZoPP2C61,ZoPP2C40,ZoPP2C37,ZoPP2C67,ZoPP2C17,ZoPP2C32,ZoPP2C87,ZoPP2C81,ZoPP2C80,ZoPP2C94 |
| MIKC | ZoPP2C27,ZoPP2C49,ZoPP2C41,ZoPP2C32,ZoPP2C2,ZoPP2C3,ZoPP2C20,ZoPP2C60,ZoPP2C14,ZoPP2C54,ZoPP2C61,ZoPP2C84,ZoPP2C12,ZoPP2C53,ZoPP2C78,ZoPP2C81,ZoPP2C9,ZoPP2C90,ZoPP2C44,ZoPP2C47,ZoPP2C73,ZoPP2C86,ZoPP2C48,ZoPP2C31,ZoPP2C4,ZoPP2C25,ZoPP2C85,ZoPP2C57,ZoPP2C17,ZoPP2C70,ZoPP2C43,ZoPP2C63,ZoPP2C79,ZoPP2C42,ZoPP2C35,ZoPP2C38,ZoPP2C40,ZoPP2C37,ZoPP2C59,ZoPP2C58,ZoPP2C36,ZoPP2C46,ZoPP2C45,ZoPP2C7,ZoPP2C16,ZoPP2C91,ZoPP2C11,ZoPP2C71,ZoPP2C67,ZoPP2C26,ZoPP2C74,ZoPP2C77,ZoPP2C50,ZoPP2C72,ZoPP2C75,ZoPP2C19,ZoPP2C92,ZoPP2C1,ZoPP2C96,ZoPP2C88,ZoPP2C34,ZoPP2C76,ZoPP2C23,ZoPP2C24,ZoPP2C94,ZoPP2C30,ZoPP2C39,ZoPP2C93,ZoPP2C87,ZoPP2C51,ZoPP2C52,ZoPP2C29,ZoPP2C21,ZoPP2C64,ZoPP2C66,ZoPP2C22,ZoPP2C33,ZoPP2C28,ZoPP2C55,ZoPP2C82,ZoPP2C10,ZoPP2C62,ZoPP2C18,ZoPP2C89,ZoPP2C56,ZoPP2C13,ZoPP2C65,ZoPP2C6,ZoPP2C15,ZoPP2C8,ZoPP2C69,ZoPP2C5,ZoPP2C68,ZoPP2C95,ZoPP2C83,ZoPP2C97,ZoPP2C80 |
| MYB | ZoPP2C49,ZoPP2C48,ZoPP2C79,ZoPP2C69,ZoPP2C25,ZoPP2C3,ZoPP2C1,ZoPP2C73,ZoPP2C86,ZoPP2C85,ZoPP2C47,ZoPP2C11,ZoPP2C50,ZoPP2C29,ZoPP2C67,ZoPP2C90,ZoPP2C27,ZoPP2C81,ZoPP2C74,ZoPP2C76,ZoPP2C60,ZoPP2C36,ZoPP2C53,ZoPP2C54,ZoPP2C9,ZoPP2C18,ZoPP2C2,ZoPP2C44,ZoPP2C72,ZoPP2C63,ZoPP2C83,ZoPP2C61,ZoPP2C20,ZoPP2C17,ZoPP2C62,ZoPP2C21,ZoPP2C93,ZoPP2C10,ZoPP2C55,ZoPP2C71,ZoPP2C91,ZoPP2C70,ZoPP2C65,ZoPP2C66,ZoPP2C88,ZoPP2C56,ZoPP2C80,ZoPP2C32,ZoPP2C89,ZoPP2C51,ZoPP2C4,ZoPP2C52,ZoPP2C5,ZoPP2C59,ZoPP2C41,ZoPP2C6,ZoPP2C31,ZoPP2C16,ZoPP2C75,ZoPP2C45,ZoPP2C26,ZoPP2C35,ZoPP2C34,ZoPP2C78,ZoPP2C58,ZoPP2C30,ZoPP2C24,ZoPP2C19,ZoPP2C42,ZoPP2C43,ZoPP2C37,ZoPP2C97,ZoPP2C95,ZoPP2C82,ZoPP2C77,ZoPP2C28,ZoPP2C33,ZoPP2C13,ZoPP2C14,ZoPP2C92,ZoPP2C38,ZoPP2C8,ZoPP2C7,ZoPP2C94,ZoPP2C40,ZoPP2C15,ZoPP2C64,ZoPP2C96,ZoPP2C23,ZoPP2C87,ZoPP2C46,ZoPP2C39,ZoPP2C57,ZoPP2C22,ZoPP2C12,ZoPP2C84,ZoPP2C68 |
| NAC | ZoPP2C1,ZoPP2C45,ZoPP2C62,ZoPP2C47,ZoPP2C49,ZoPP2C58,ZoPP2C32,ZoPP2C25,ZoPP2C27,ZoPP2C65,ZoPP2C66,ZoPP2C13,ZoPP2C8,ZoPP2C36,ZoPP2C96,ZoPP2C19,ZoPP2C79,ZoPP2C6,ZoPP2C41,ZoPP2C82,ZoPP2C70,ZoPP2C81,ZoPP2C59,ZoPP2C78,ZoPP2C54,ZoPP2C11,ZoPP2C95,ZoPP2C68,ZoPP2C84,ZoPP2C17,ZoPP2C46,ZoPP2C94,ZoPP2C60,ZoPP2C5,ZoPP2C61,ZoPP2C18,ZoPP2C24,ZoPP2C74,ZoPP2C86,ZoPP2C64,ZoPP2C3,ZoPP2C22,ZoPP2C29,ZoPP2C85,ZoPP2C91,ZoPP2C4,ZoPP2C53,ZoPP2C63,ZoPP2C2,ZoPP2C51,ZoPP2C90,ZoPP2C15,ZoPP2C55,ZoPP2C42,ZoPP2C20,ZoPP2C56,ZoPP2C52,ZoPP2C26,ZoPP2C72,ZoPP2C31,ZoPP2C21,ZoPP2C83,ZoPP2C88,ZoPP2C93,ZoPP2C16,ZoPP2C10,ZoPP2C67,ZoPP2C50,ZoPP2C77,ZoPP2C30,ZoPP2C71,ZoPP2C34,ZoPP2C87,ZoPP2C89,ZoPP2C14,ZoPP2C7,ZoPP2C76,ZoPP2C69,ZoPP2C48,ZoPP2C38,ZoPP2C35,ZoPP2C92,ZoPP2C43,ZoPP2C28,ZoPP2C75,ZoPP2C80,ZoPP2C39,ZoPP2C57,ZoPP2C12,ZoPP2C40,ZoPP2C37,ZoPP2C33,ZoPP2C9,ZoPP2C73,ZoPP2C97,ZoPP2C23,ZoPP2C44 |
| NLP | ZoPP2C88,ZoPP2C90,ZoPP2C66,ZoPP2C16,ZoPP2C72,ZoPP2C6,ZoPP2C27,ZoPP2C87,ZoPP2C25,ZoPP2C55,ZoPP2C50,ZoPP2C53,ZoPP2C95,ZoPP2C49,ZoPP2C64,ZoPP2C47,ZoPP2C79,ZoPP2C1,ZoPP2C18,ZoPP2C5,ZoPP2C38,ZoPP2C42,ZoPP2C4,ZoPP2C29,ZoPP2C7,ZoPP2C12,ZoPP2C85,ZoPP2C60,ZoPP2C70,ZoPP2C61,ZoPP2C13,ZoPP2C2,ZoPP2C84,ZoPP2C69,ZoPP2C80,ZoPP2C30,ZoPP2C78,ZoPP2C94,ZoPP2C44,ZoPP2C21,ZoPP2C81,ZoPP2C62,ZoPP2C76,ZoPP2C86,ZoPP2C3,ZoPP2C45,ZoPP2C36,ZoPP2C40,ZoPP2C37,ZoPP2C11,ZoPP2C32,ZoPP2C19,ZoPP2C92,ZoPP2C63,ZoPP2C31,ZoPP2C48,ZoPP2C24 |
| RAV | ZoPP2C74,ZoPP2C59,ZoPP2C78,ZoPP2C70,ZoPP2C42,ZoPP2C89,ZoPP2C27,ZoPP2C20,ZoPP2C10,ZoPP2C52,ZoPP2C29,ZoPP2C12,ZoPP2C79,ZoPP2C40,ZoPP2C81,ZoPP2C19,ZoPP2C25,ZoPP2C16,ZoPP2C85,ZoPP2C31,ZoPP2C36,ZoPP2C49,ZoPP2C30,ZoPP2C48,ZoPP2C95,ZoPP2C56,ZoPP2C26,ZoPP2C86,ZoPP2C62,ZoPP2C63,ZoPP2C94,ZoPP2C55,ZoPP2C65,ZoPP2C66,ZoPP2C92,ZoPP2C71,ZoPP2C6,ZoPP2C61,ZoPP2C80,ZoPP2C53,ZoPP2C38,ZoPP2C44,ZoPP2C51,ZoPP2C50,ZoPP2C21,ZoPP2C24,ZoPP2C4,ZoPP2C47,ZoPP2C90,ZoPP2C76,ZoPP2C75,ZoPP2C72,ZoPP2C41,ZoPP2C18,ZoPP2C82,ZoPP2C60,ZoPP2C2,ZoPP2C13,ZoPP2C11,ZoPP2C67,ZoPP2C1,ZoPP2C58,ZoPP2C84,ZoPP2C23,ZoPP2C37 |
| SBP | ZoPP2C63,ZoPP2C1,ZoPP2C81,ZoPP2C3,ZoPP2C36,ZoPP2C2,ZoPP2C27,ZoPP2C35,ZoPP2C26,ZoPP2C7,ZoPP2C40,ZoPP2C37,ZoPP2C90,ZoPP2C85,ZoPP2C9,ZoPP2C15,ZoPP2C8,ZoPP2C79,ZoPP2C91,ZoPP2C76,ZoPP2C4,ZoPP2C84,ZoPP2C31,ZoPP2C75,ZoPP2C47,ZoPP2C86,ZoPP2C94,ZoPP2C61,ZoPP2C49,ZoPP2C78,ZoPP2C32,ZoPP2C30,ZoPP2C74,ZoPP2C60,ZoPP2C18,ZoPP2C42,ZoPP2C83,ZoPP2C58,ZoPP2C53,ZoPP2C73,ZoPP2C21,ZoPP2C28,ZoPP2C41,ZoPP2C46,ZoPP2C50,ZoPP2C11,ZoPP2C71,ZoPP2C87,ZoPP2C14,ZoPP2C93,ZoPP2C19,ZoPP2C88,ZoPP2C55,ZoPP2C10,ZoPP2C22,ZoPP2C43,ZoPP2C67,ZoPP2C82,ZoPP2C25,ZoPP2C29,ZoPP2C45,ZoPP2C33,ZoPP2C16,ZoPP2C57,ZoPP2C89,ZoPP2C48,ZoPP2C77,ZoPP2C44,ZoPP2C12,ZoPP2C62,ZoPP2C72,ZoPP2C17,ZoPP2C38,ZoPP2C59,ZoPP2C92,ZoPP2C54 |
| SRS | ZoPP2C76,ZoPP2C49,ZoPP2C21,ZoPP2C59,ZoPP2C27,ZoPP2C73,ZoPP2C6,ZoPP2C63,ZoPP2C86,ZoPP2C10,ZoPP2C45,ZoPP2C31,ZoPP2C74,ZoPP2C19,ZoPP2C85,ZoPP2C18,ZoPP2C2,ZoPP2C1,ZoPP2C30,ZoPP2C71,ZoPP2C96,ZoPP2C88,ZoPP2C46,ZoPP2C36,ZoPP2C93 |
| TALE | ZoPP2C32,ZoPP2C47,ZoPP2C4,ZoPP2C76,ZoPP2C96,ZoPP2C30,ZoPP2C31,ZoPP2C25,ZoPP2C85,ZoPP2C27,ZoPP2C86,ZoPP2C95,ZoPP2C49,ZoPP2C34,ZoPP2C87,ZoPP2C80,ZoPP2C50,ZoPP2C71,ZoPP2C14,ZoPP2C51,ZoPP2C91,ZoPP2C79,ZoPP2C23,ZoPP2C73,ZoPP2C10,ZoPP2C28,ZoPP2C15,ZoPP2C8,ZoPP2C84,ZoPP2C13,ZoPP2C93,ZoPP2C44,ZoPP2C17,ZoPP2C22,ZoPP2C46,ZoPP2C43,ZoPP2C82,ZoPP2C90,ZoPP2C59,ZoPP2C56,ZoPP2C61,ZoPP2C89,ZoPP2C35,ZoPP2C66,ZoPP2C65,ZoPP2C69,ZoPP2C48,ZoPP2C97,ZoPP2C88,ZoPP2C57,ZoPP2C36,ZoPP2C2,ZoPP2C67,ZoPP2C60,ZoPP2C7,ZoPP2C33,ZoPP2C68,ZoPP2C70,ZoPP2C26,ZoPP2C19,ZoPP2C20,ZoPP2C83,ZoPP2C3,ZoPP2C5,ZoPP2C,ZoPP2C72,ZoPP2C74,ZoPP2C11,ZoPP2C29,ZoPP2C77,ZoPP2C16,ZoPP2C1,ZoPP2C9,ZoPP2C94,ZoPP2C53,ZoPP2C39,ZoPP2C42,ZoPP2C64,ZoPP2C81,ZoPP2C52,ZoPP2C24,ZoPP2C12,ZoPP2C38,ZoPP2C45,ZoPP2C6,ZoPP2C41,ZoPP2C58,ZoPP2C21,ZoPP2C92,ZoPP2C55,ZoPP2C63,ZoPP2C54 |
| TCP | ZoPP2C93,ZoPP2C44,ZoPP2C4,ZoPP2C47,ZoPP2C27,ZoPP2C83,ZoPP2C97,ZoPP2C30,ZoPP2C70,ZoPP2C53,ZoPP2C3,ZoPP2C2,ZoPP2C1,ZoPP2C29,ZoPP2C56,ZoPP2C57,ZoPP2C61,ZoPP2C76,ZoPP2C54,ZoPP2C32,ZoPP2C49,ZoPP2C79,ZoPP2C90,ZoPP2C72,ZoPP2C26,ZoPP2C86,ZoPP2C59,ZoPP2C67,ZoPP2C55,ZoPP2C48,ZoPP2C46,ZoPP2C25,ZoPP2C85,ZoPP2C89,ZoPP2C15,ZoPP2C8,ZoPP2C78,ZoPP2C81,ZoPP2C94,ZoPP2C95,ZoPP2C50,ZoPP2C82,ZoPP2C23,ZoPP2C31,ZoPP2C87,ZoPP2C60,ZoPP2C63,ZoPP2C18,ZoPP2C45,ZoPP2C73,ZoPP2C62,ZoPP2C58,ZoPP2C75,ZoPP2C16,ZoPP2C28,ZoPP2C7,ZoPP2C12,ZoPP2C24,ZoPP2C19,ZoPP2C11,ZoPP2C42,ZoPP2C69,ZoPP2C80,ZoPP2C20,ZoPP2C36,ZoPP2C41,ZoPP2C33,ZoPP2C17,ZoPP2C84,ZoPP2C9,ZoPP2C40,ZoPP2C74,ZoPP2C2,ZoPP2C39,ZoPP2C52,ZoPP2C88,ZoPP2C68,ZoPP2C71,ZoPP2C91 |
| Trihelix | ZoPP2C32,ZoPP2C56,ZoPP2C43,ZoPP2C30,ZoPP2C8,ZoPP2C52,ZoPP2C68,ZoPP2C73,ZoPP2C29,ZoPP2C27,ZoPP2C11,ZoPP2C72,ZoPP2C12,ZoPP2C31,ZoPP2C60,ZoPP2C63,ZoPP2C15,ZoPP2C93,ZoPP2C9,ZoPP2C95,ZoPP2C85,ZoPP2C76,ZoPP2C74,ZoPP2C89,ZoPP2C38,ZoPP2C84,ZoPP2C86,ZoPP2C49,ZoPP2C10,ZoPP2C28,ZoPP2C82,ZoPP2C91,ZoPP2C34,ZoPP2C26,ZoPP2C96,ZoPP2C90,ZoPP2C75,ZoPP2C18,ZoPP2C64,ZoPP2C97,ZoPP2C92,ZoPP2C33,ZoPP2C87,ZoPP2C70,ZoPP2C19,ZoPP2C69,ZoPP2C61,ZoPP2C83,ZoPP2C94,ZoPP2C53,ZoPP2C50,ZoPP2C14,ZoPP2C25,ZoPP2C35,ZoPP2C20,ZoPP2C22,ZoPP2C57,ZoPP2C6,ZoPP2C48,ZoPP2C21,ZoPP2C40,ZoPP2C37,ZoPP2C80,ZoPP2C71,ZoPP2C3,ZoPP2C17,ZoPP2C16,ZoPP2C47,ZoPP2C59,ZoPP2C41,ZoPP2C42,ZoPP2C39,ZoPP2C4,ZoPP2C67,ZoPP2C81,ZoPP2C44,ZoPP2C79,ZoPP2C65,ZoPP2C66,ZoPP2C1,ZoPP2C7,ZoPP2C62,ZoPP2C23,ZoPP2C46,ZoPP2C45,ZoPP2C51,ZoPP2C36,ZoPP2C88,ZoPP2C13,ZoPP2C5,ZoPP2C58,ZoPP2C55,ZoPP2C54,ZoPP2C2,ZoPP2C78 |
| WOX | ZoPP2C27,ZoPP2C44,ZoPP2C11,ZoPP2C49,ZoPP2C72,ZoPP2C59,ZoPP2C32,ZoPP2C79,ZoPP2C45,ZoPP2C46,ZoPP2C19,ZoPP2C36,ZoPP2C62,ZoPP2C85,ZoPP2C92,ZoPP2C41,ZoPP2C24,ZoPP2C74,ZoPP2C47,ZoPP2C88,ZoPP2C43,ZoPP2C14,ZoPP2C55,ZoPP2C67,ZoPP2C28,ZoPP2C10,ZoPP2C20,ZoPP2C94,ZoPP2C29,ZoPP2C76,ZoPP2C75,ZoPP2C7,ZoPP2C16,ZoPP2C70,ZoPP2C89,ZoPP2C1,ZoPP2C81,ZoPP2C61,ZoPP2C17,ZoPP2C53,ZoPP2C86,ZoPP2C31,ZoPP2C50,ZoPP2C9,ZoPP2C87,ZoPP2C21,ZoPP2C3,ZoPP2C48,ZoPP2C25,ZoPP2C26,ZoPP2C63,ZoPP2C52,ZoPP2C40,ZoPP2C37,ZoPP2C4,ZoPP2C2,ZoPP2C90,ZoPP2C60,ZoPP2C56,ZoPP2C38,ZoPP2C66,ZoPP2C73,ZoPP2C93,ZoPP2C18,ZoPP2C34,ZoPP2C65,ZoPP2C42,ZoPP2C5,ZoPP2C78,ZoPP2C54,ZoPP2C35,ZoPP2C12,ZoPP2C84,ZoPP2C51,ZoPP2C58,ZoPP2C71,ZoPP2C96,ZoPP2C77,ZoPP2C80,ZoPP2C68 |
| WRKY | ZoPP2C46,ZoPP2C65,ZoPP2C66,ZoPP2C63,ZoPP2C27,ZoPP2C85,ZoPP2C31,ZoPP2C76,ZoPP2C39,ZoPP2C86,ZoPP2C13,ZoPP2C7,ZoPP2C36,ZoPP2C42,ZoPP2C47,ZoPP2C40,ZoPP2C37,ZoPP2C16,ZoPP2C58,ZoPP2C55,ZoPP2C96,ZoPP2C11,ZoPP2C64,ZoPP2C90,ZoPP2C1,ZoPP2C5,ZoPP2C88,ZoPP2C84,ZoPP2C93,ZoPP2C87,ZoPP2C25,ZoPP2C70,ZoPP2C57,ZoPP2C92,ZoPP2C73,ZoPP2C49,ZoPP2C81,ZoPP2C41,ZoPP2C45,ZoPP2C38,ZoPP2C6,ZoPP2C69,ZoPP2C3,ZoPP2C79,ZoPP2C67,ZoPP2C60,ZoPP2C3,ZoPP2C71,ZoPP2C4,ZoPP2C80,ZoPP2C97,ZoPP2C59,ZoPP2C44,ZoPP2C94,ZoPP2C17,ZoPP2C61,ZoPP2C89,ZoPP2C72,ZoPP2C19,ZoPP2C54,ZoPP2C29,ZoPP2C21,ZoPP2C14,ZoPP2C43,ZoPP2C48,ZoPP2C62,ZoPP2C53,ZoPP2C12,ZoPP2C75,ZoPP2C26,ZoPP2C95,ZoPP2C78,ZoPP2C83,ZoPP2C15,ZoPP2C8,ZoPP2C30,ZoPP2C50,ZoPP2C18,ZoPP2C20,ZoPP2C56,ZoPP2C2,ZoPP2C74,ZoPP2C91,ZoPP2C52,ZoPP2C82 |
| YABBY | ZoPP2C36,ZoPP2C27,ZoPP2C20,ZoPP2C32,ZoPP2C44,ZoPP2C76,ZoPP2C47,ZoPP2C49,ZoPP2C14,ZoPP2C1,ZoPP2C89,ZoPP2C13,ZoPP2C72,ZoPP2C87,ZoPP2C17,ZoPP2C3,ZoPP2C4,ZoPP2C19,ZoPP2C25,ZoPP2C63,ZoPP2C40,ZoPP2C37,ZoPP2C2,ZoPP2C16,ZoPP2C79,ZoPP2C71,ZoPP2C10,ZoPP2C97,ZoPP2C54,ZoPP2C92,ZoPP2C59,ZoPP2C60,ZoPP2C11,ZoPP2C6,ZoPP2C67,ZoPP2C55,ZoPP2C50,ZoPP2C18,ZoPP2C74,ZoPP2C70,ZoPP2C48,ZoPP2C85,ZoPP2C81,ZoPP2C65,ZoPP2C66,ZoPP2C94,ZoPP2C9,ZoPP2C15,ZoPP2C8,ZoPP2C83,ZoPP2C53,ZoPP2C46,ZoPP2C34,ZoPP2C30,ZoPP2C73,ZoPP2C75,ZoPP2C78,ZoPP2C61,ZoPP2C29,ZoPP2C86,ZoPP2C45,ZoPP2C90,ZoPP2C41,ZoPP2C38,ZoPP2C58,ZoPP2C68,ZoPP2C93 |

Table S8 The number of transcription factor binding sites

| TF family | ZoPP2C binding site |
| --- | --- |
| ERF | 9351 |
| Dof | 5158 |
| C2H2 | 3229 |
| MIKC | 3174 |
| MYB | 2853 |
| NAC | 2400 |
| HD-ZIP | 1817 |
| WRKY | 1689 |
| BBR-BPC | 1563 |
| G2-like | 1476 |
| AP2 | 1418 |
| GATA | 1330 |
| HSF | 1059 |
| bZIP | 1033 |
| bHLH | 798 |
| TALE | 676 |
| TCP | 595 |
| ARF | 572 |
| Trihelix | 533 |
| WOX | 531 |
| C3H | 530 |
| CPP | 504 |
| B3 | 497 |
| SBP | 251 |
| YABBY | 237 |
| EIL | 216 |
| ARR-B | 133 |
| BES1 | 128 |
| Nin-like | 110 |
| RAV | 104 |
| LBD | 79 |
| SRS | 49 |
| E2F/DP | 48 |
| CAMTA | 32 |
| LFY | 16 |

Table S9 Source of data sets of transcriptomes of ginger

| Accession ID | Description | Data size |
| --- | --- | --- |
| PRJNA788194 | Developmental process of ginger rhizome, mechanism of color formation during growth of ginger, 3 biological replicates | Unknown |
| PRJNA592215 | Four samples of ginger red stem interior, green stem, yellow rhizome and red stem surface, 3 biological replicates | 254M |
| PRJNA380847 | Ginger rhizomes were treated with sterile water and *Fusarium solani* respectively for 3d, 3 biological replicates | Unknown |
| PRJNA911443 | Low temperature treatment of Ginger Seedling, 3 biological replicates | 16.8G |
