# Supplementary figures and images for: Genome-wide analysis and expression pattern of the ZoPP2C gene family in Zingiber officinale Roscoe
Source: BMC Genomics. 2024 Jan 20;25:83. doi: 10.1186/s12864-024-09966-w (PMC10799369; doi:10.1186/s12864-024-09966-w)

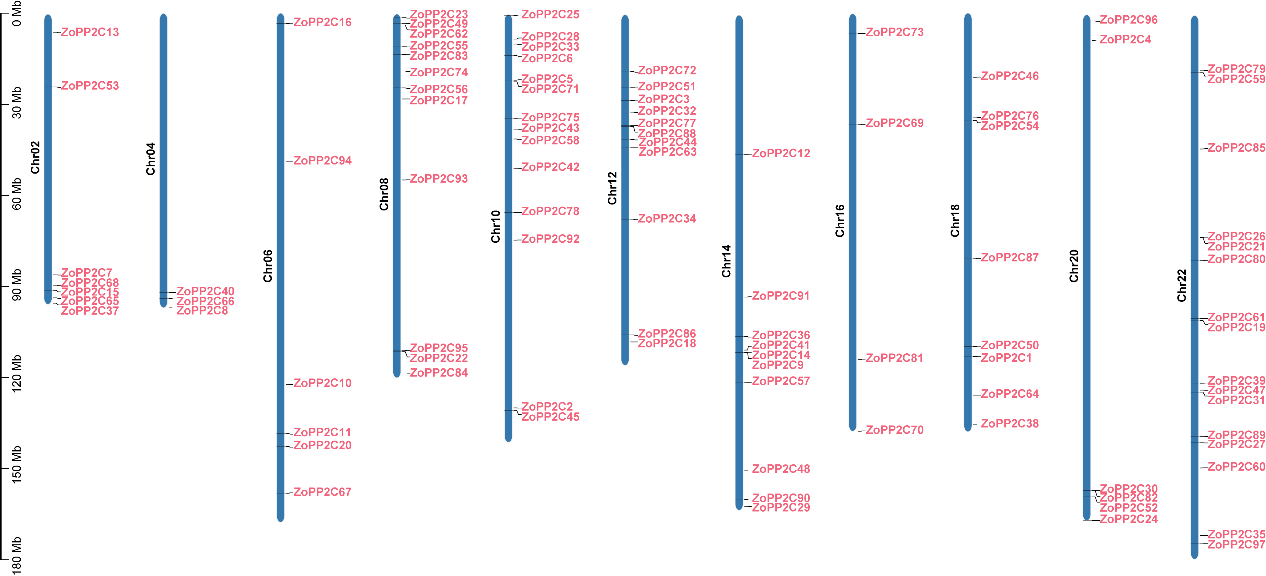


**Fig. S1** ZoPP2C gene chromosomal localization.


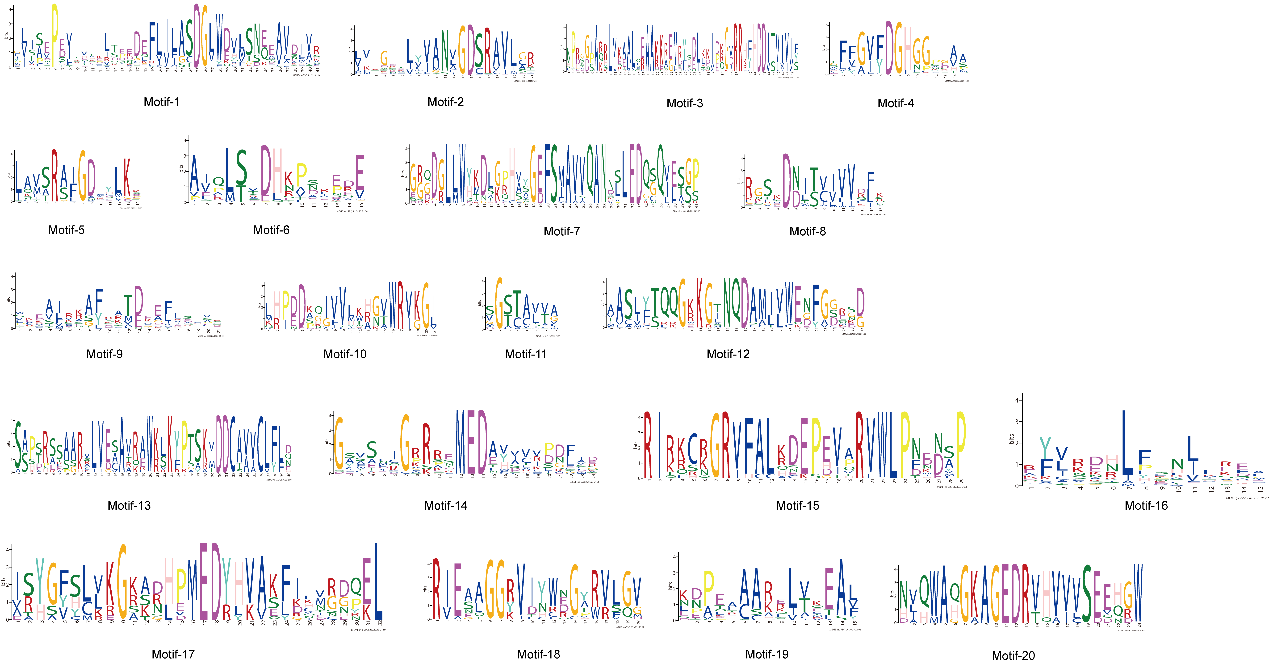


**Fig. S2** 1-20 motif sequence logos.

Supplement: Supplementary file 2 — Additional file 2: Fig. S1. ZoPP2C gene chromosomal localization. Fig. S2. 1-20 motif sequence logos. [file 12864_2024_9966_MOESM2_ESM.docx]
